# Comparative Genetic Analysis of Psoriatic Arthritis and Psoriasis for the Discovery of Genetic Risk Factors and Risk Prediction Modeling

**DOI:** 10.1002/art.42154

**Published:** 2022-08-04

**Authors:** Mehreen Soomro, Michael Stadler, Nick Dand, James Bluett, Deepak Jadon, Farideh Jalali‐najafabadi, Michael Duckworth, Pauline Ho, Helena Marzo‐Ortega, Philip S. Helliwell, Anthony W. Ryan, David Kane, Eleanor Korendowych, Michael A. Simpson, Jonathan Packham, Ross McManus, Cem Gabay, Céline Lamacchia, Michael J. Nissen, Matthew A. Brown, Suzanne M. M. Verstappen, Tjeerd Van Staa, Jonathan N. Barker, Catherine H. Smith, the BADBIR Study Group, Robert Chalmers, Carsten Flohr, Karen Watson, David Prieto‐Merino, Oras Alabas, Jonathan Barker, Gabrielle Becher, Anthony Bewley, David Burden, Simon Morrison, Phil Laws, Ian Evans, Christopher Griffiths, Shehnaz Ahmed, Brian Kirby, Elise Kleyn, Linda Lawson, Teena Mackenzie, Tess McPherson, Kathleen McElhone, Ruth Murphy, Anthony Ormerod, Caroline Owen, Nick Reynolds, Amir Rashid, Catherine Smith, Richard Warren, the BSTOP study group, David Burden, Catherine Smith, Stefan Siebert, Sara Brown, Helen McAteer, Julia Schofield, Nick Dand, Oliver FitzGerald, Neil McHugh, Richard B. Warren, John Bowes, Anne Barton

**Affiliations:** ^1^ Centre for Genetics and Genomics Versus Arthritis, Centre for Musculoskeletal Research, Manchester Academic Health Science Centre The University of Manchester Manchester UK; ^2^ King's College London London UK; ^3^ Centre for Genetics and Genomics Versus Arthritis, Centre for Musculoskeletal Research, Manchester Academic Health Science Center, The University of Manchester, NIHR Manchester Musculoskeletal Biomedical Research Unit, Manchester University NHS Foundation Trust Manchester UK; ^4^ University of Cambridge Cambridge UK; ^5^ St John's Institute of Dermatology, King's College London London UK; ^6^ NIHR Leeds Biomedical Research Centre, Leeds Teaching Hospitals Trust, Leeds Institute of Rheumatic and Musculoskeletal Medicine, University of Leeds Leeds UK; ^7^ Trinity Translational Medicine Institute, Trinity College Dublin and Genuity Science Dublin Ireland; ^8^ Tallaght University Hospital and Trinity College Dublin Dublin Ireland; ^9^ Royal National Hospital for Rheumatic Diseases and University of Bath Bath UK; ^10^ Haywood Hospital and Midlands Partnership NHS Foundation Trust, Stoke on Trent, UK, and University of Nottingham Nottingham UK; ^11^ Trinity Translational Medicine Institute, Trinity College Dublin Dublin Ireland; ^12^ Geneva University Hospitals, University of Geneva Geneva Switzerland; ^13^ Geneva University Hospitals Geneva Switzerland; ^14^ King's College London and Genomics England London UK; ^15^ NIHR Manchester Musculoskeletal Biomedical Research Unit Manchester University NHS Foundation Trust, Manchester Academic Health Science Centre, Centre for Epidemiology Versus Arthritis, Centre for Musculoskeletal Research, University of Manchester Manchester UK; ^16^ Health e‐Research Centre, Health Data Research UK North, University of Manchester Manchester UK; ^17^ St John's Institute of Dermatology, Guy's and St Thomas’ NHS Foundation Trust and King's College London London UK; ^18^ Conway Institute of Biomolecular and Biomedical Research, University College Dublin Dublin Ireland; ^19^ Royal National Hospital for Rheumatic Diseases, University of Bath Bath UK; ^20^ Dermatology Centre, Salford Royal NHS Foundation Trust, Manchester NIHR Biomedical Research Centre, University of Manchester Manchester UK

## Abstract

**Objectives:**

Psoriatic arthritis (PsA) has a strong genetic component, and the identification of genetic risk factors could help identify the ~30% of psoriasis patients at high risk of developing PsA. Our objectives were to identify genetic risk factors and pathways that differentiate PsA from cutaneous‐only psoriasis (PsC) and to evaluate the performance of PsA risk prediction models.

**Methods:**

Genome‐wide meta‐analyses were conducted separately for 5,065 patients with PsA and 21,286 healthy controls and separately for 4,340 patients with PsA and 6,431 patients with PsC. The heritability of PsA was calculated as a single‐nucleotide polymorphism (SNP)–based heritability estimate (h^2^_SNP_) and biologic pathways that differentiate PsA from PsC were identified using Priority Index software. The generalizability of previously published PsA risk prediction pipelines was explored, and a risk prediction model was developed with external validation.

**Results:**

We identified a novel genome‐wide significant susceptibility locus for the development of PsA on chromosome 22q11 (rs5754467; *P* = 1.61 × 10^−9^), and key pathways that differentiate PsA from PsC, including NF‐κB signaling (adjusted *P* = 1.4 × 10^−45^) and Wnt signaling (adjusted *P* = 9.5 × 10^−58^). The heritability of PsA in this cohort was found to be moderate (h^2^_SNP_ = 0.63), which was similar to the heritability of PsC (h^2^_SNP_ = 0.61). We observed modest performance of published classification pipelines (maximum area under the curve 0.61), with similar performance of a risk model derived using the current data.

**Conclusion:**

Key biologic pathways associated with the development of PsA were identified, but the investigation of risk classification revealed modest utility in the available data sets, possibly because many of the PsC patients included in the present study were receiving treatments that are also effective in PsA. Future predictive models of PsA should be tested in PsC patients recruited from primary care.

## INTRODUCTION

Psoriatic arthritis (PsA) is a chronic inflammatory condition characterized by the presence of peripheral arthritis, dactylitis, enthesitis, and axial spondyloarthritis (1). PsA affects between 14% and 30% of patients with psoriasis, leading to significant disability and a reduced quality of life (1, 2, 3). The ability to identify patients with psoriasis who are at a high risk of developing PsA is an important goal for clinical research, as this would allow early intervention to reduce the impact of PsA and ultimately lead to preventative treatments.

PsA is a typical complex disease in which susceptibility is influenced by a combination of environmental, lifestyle, and genetic risk factors. Previous family pedigree studies have estimated that the heritability of PsA far exceeds that of psoriasis alone, providing evidence of an increased genetic component which, once identified, could help to differentiate those patients at high risk of developing PsA by inclusion of genetic risk factors in clinical prediction models (4, 5, 6). However, the results of these family studies have been challenged by data from large‐scale case–control studies analyzing variations in single‐nucleotide polymorphisms (SNPs), in which only limited differences in heritability estimates have been demonstrated between patients with PsA and patients with psoriasis (7). Several studies have identified genetic risk factors that are specific to PsA, including amino acids within HLA–B and variants at the *IL23R* gene, and the current aim is to translate these genetic discoveries into improved clinical outcomes (8, 9, 10, 11, 12). A recent study demonstrated high performance in accurately distinguishing PsA from cutaneous‐only psoriasis (PsC) using prediction models based on genetic risk factors. Although this study demonstrated validity by internal cross‐validation methods, assessment of these models for generalizability in external data sets is still warranted (13).

To help further our understanding of the genetic basis for PsA, we have constructed a large integrated genetic data set of PsA patients, PsC patients, and population controls imputed to the latest population reference panels. We supplemented this data set by performing a meta‐analysis using UK Biobank data, allowing us to contrast PsA patients with population controls and PsC patients, and to explore differences in the genetic architecture of the 2 traits that could explain the progression to PsA. These data can be used to further our understanding of key genes and biologic pathways important in psoriatic disease using state‐of‐the‐art bioinformatics tools and could be further used to explore the utility of genetic risk prediction models for classifying PsA.

## PATIENTS AND METHODS

### PsA genome‐wide association study (GWAS) cohort

A total of 4,072 patients with PsA were recruited from rheumatology centers in the UK, Ireland, and Switzerland, from the prospective Swiss Clinical Quality Management (SCQM) registry, and from Australia. Patients recruited in Manchester were diagnosed by a rheumatologist based on the presence of both psoriasis and inflammatory peripheral arthritis, regardless of rheumatoid factor status. While the majority of patients satisfied the CASPAR (Classification of Psoriatic Arthritis) classification system (14), some were recruited prior to the introduction of this classification system. All patients provided written informed consent (UK PsA National Repository Multicentre Research Ethics Committee reference no. 99/8/84). Samples from the Axial Disease in Psoriatic Arthritis Study (ADIPSA) cohort were collected with ethics approval from the French Regional Ethics Committee (reference no. 12/SW/0110). The Leeds cohort comprises adult patients with a clinical diagnosis of PsA fulfilling the CASPAR classification criteria who were recruited as part of an in‐house biobank study investigating SNPs of immune response genes in patients with psoriasis, patients with PsA, and patients with ankylosing spondylitis and their relationship to disease susceptibility, articular and extraarticular manifestations, and response to treatment (Research Ethics Committee reference no. 04/Q1205/65, IRAS project no. 232680). All patients provided written informed consent.

A total of 283 patients with PsA were recruited from St. Vincent's University Hospital observational PsA cohort. All patients met the CASPAR criteria. The study protocol received approval from the local ethics committee of St. Vincent's University Hospital. Written informed consent was obtained from all patients. In addition, a total of 272 patients with PsA were recruited from the prospective SCQM registry in which diagnosis was based on the CASPAR criteria. The study protocol received approval from the local ethics committee of the University Hospital of Geneva (protocol no. 10‐089) and the SCQM Biobank Scientific Advisory Board and followed the Guidelines for Good Clinical Practice. Written informed consent was obtained from all patients. A summary of available clinical phenotype data for this cohort is given in Supplementary Table 1 (available on the *Arthritis & Rheumatology* website at http://onlinelibrary.wiley.com/doi/10.1002/art.42154).

### Psoriasis GWAS cohort

We had access to data from 2,086 psoriasis patient samples obtained through the Biomarkers of Systemic Treatment Outcomes in Psoriasis study (BSTOP) described previously (9). Analysis of patients was restricted to those with no previous diagnosis of PsA, and we refer to this sample group as having cutaneous‐only psoriasis (PsC). Patients with psoriasis requiring systemic therapy who also consented to enrolment in the British Association of Dermatologists Biologics Interventions Registry (a UK pharmacovigilance registry) were recruited to BSTOP from over 60 secondary and tertiary care outpatient dermatology departments throughout the UK including centers in London, Manchester, Nottingham, and Liverpool. All patients provided written informed consent (BSTOP Ethics reference no. 11/H0802/7). Classification of PsC in the BSTOP cohort is based on information collected at multiple follow‐up consultancies (one every 6 months in the first 3 years and then once annually) during which a research nurse or clinician actively investigated the patient's medical records for the presence of a PsA diagnosis made by a rheumatologist. On average, patients in this cohort had a psoriasis disease duration of 27 years without a recorded PsA diagnosis (see Supplementary Figure 1A, available at http://onlinelibrary.wiley.com/doi/10.1002/art.42154) and had been participants in the British Association of Dermatologists Biologic and Immunomodulators Register (BADBIR) study for ~7 years (Supplementary Figure 1B) with an average of 8 follow‐up consultancies.

### Control population GWAS cohort

As controls, genotype data were available for 9,965 general population subjects from the UK Household Longitudinal Study (https://www.understandingsociety.ac.uk/), accessed through the European Genotype‐phenome Archive. Samples were genotyped at the Wellcome Trust Sanger Institute using the Illumina Infinium CoreExome genotyping array. The quality control procedures applied to genotyping of control samples were consistent with those described below for patient samples.

### Genotyping and statistical quality control

PsA samples were genotyped using the Illumina Infinium CoreExome genotyping array. This was performed in accordance with the manufacturer's instructions where genotype calling was performed using the GenCall algorithm in the GenomeStudio Data Analysis software platform (Genotyping Module version 1.8.4). Psoriasis samples were genotyped using the Illumina HumanOmniExpressExome‐8v1‐2_A array performed at King's College London with quality control as previously described (15). The 3 data sets (PsA, PsC, and controls) were combined with the intersection of SNPs being retained; hereafter, this is referred as the PsA‐BSTOP GWAS data set. Further details are provided in the Supplementary Materials and Methods and Supplementary Figure 2 (available at http://onlinelibrary.wiley.com/doi/10.1002/art.42154).

### Imputation

Imputation was performed for the combined data set of PsA, PsC, and control samples described above. Prior to imputation, SNPs with ambiguous alleles (C/G and A/T) were excluded, and remaining SNPs were aligned to the Haplotype Reference Consortium (HRC) panel (version 1.1) using the HRC imputation preparation tool (https://www.well.ox.ac.uk/~wrayner/tools/). Imputation was performed using the Michigan Imputation server in which phasing was performed with Shapeit2 and imputation was performed with the HRC panel. Following imputation, SNPs were excluded based on a minor allele frequency (MAF) of <0.01 and imputation accuracy of r^2^ <0.5.

### UK Biobank

We accessed imputed genotype data from the UK Biobank (application number 799) for self‐reported outcomes in 731 PsA patients and 3,197 psoriasis patients (16). Control population data were obtained using random sampling from the remaining cohort at a ratio of 4 controls to 1 patient to minimize inflation of test statistics due to case–control imbalance. All participants were selected from the subset of White patients of British ancestry. In addition, we created a data set based on International Statistical Classification of Diseases and Related Health Problems, Tenth Revision (ICD‐10) codes L40 and L405, which yielded a cohort of 795 psoriasis patients and 435 PsA patients.

### PsA Immunochip data set

Genotype data were available for 1,962 PsA patients and 8,923 controls (controls were recruited from the 1958 Birth Cohort and the National Blood Service) (17). Sample overlap with the GWAS and UK Biobank data sets was determined using identity by descent analysis (Kinship‐based Inference for GWAS software) and duplicate samples were excluded from the Immunochip data set, leaving a total of 725 PsA patients and 8,897 controls.

### Association testing and meta‐analysis

Case–control association analyses were performed with the SNPTEST software package (version 2.5.2) using their scoring method to account for imputation uncertainty. Meta‐analyses were conducted using an inverse variance meta‐analysis assuming fixed effects with version 2.2.2 of the software package Genome‐Wide Association Meta‐Analysis (GWAMA) (18). Lambda genomic control (λ_gc_) inflation factor, corrected for sample size (λ_gc1000_), was calculated to test for inflation of test statistics attributable to population stratification, and potential inflation of test statistics from other sources. An overview of these analyses is available in Supplementary Figure 3 and further details are provided in the Supplementary Materials and Methods (association testing and meta‐analysis) (available at http://onlinelibrary.wiley.com/doi/10.1002/art.42154).

### Heritability estimates

Heritability of PsA and PsC was estimated in the PsA‐BSTOP GWAS data set using genome‐wide complex trait analysis (GCTA software). SNPs were stratified into quartiles based on levels of linkage disequilibrium, and then further stratified into bins according to MAF values (see Supplementary Materials and Methods). Calculations were performed with no prevalence specified and with a specified disease prevalence of 1% for comparison with previously reported estimates (7). Both calculations were repeated with SNPs excluded from the major histocompatibility complex (MHC).

### Gene and pathway prioritization

We prioritized key genes and pathways for psoriatic disease using the priority index (Pi) pipeline (19). Genes were prioritized based on the following criteria: 1) proximity of SNPs to genes and localization to their topologically associated domain (cell line GM12878); 2) physical interaction determined by chromatin conformation (monocytes, macrophages [M0, M1, M2], neutrophils, CD4 T cells [naive and total], CD8 T cells [naive and total], or B cells [naive and total]); 3) correlation with gene expression (monocytes [unstimulated, lipopolysaccharide (LPS)–stimulated for 2 hours and 24 hours, interferon‐γ (IFNγ)–stimulated for 24 hours], B cells, peripheral whole blood, CD4 T cells, CD8 T cells, neutrophils, or natural killer cells). Further scoring was based on gene ontologies for immune function, immune phenotype, and rare genetic immune diseases according to the OMIM. Enrichment in pathways was based on Reactome pathways.

### Reproducing existing pipelines

A recent publication reported the performance of an analysis pipeline based on multiple machine learning approaches for the classification of PsA in patients with psoriasis, referred to hereafter as the Michigan classification pipeline (13). Based on the author recommendations for reproducing this pipeline, we trained 2 of the reported best performing machine learning algorithms (random forest and conditional inference forest) in the PsA‐BSTOP GWAS data set to capture the cohort‐specific parameters using the reported model parameters and the sets of genetics features (see Supplementary Table 2, available at http://onlinelibrary.wiley.com/doi/10.1002/art.42154). The models were internally validated using k‐fold cross‐validation and were trained using the Machine Learning in R (MLR) package (see Supplementary Figure 4A for an overview and the Supplementary Materials and Methods for further details, available at http://onlinelibrary.wiley.com/doi/10.1002/art.42154).

### Model development and validation

We developed a PsA prediction model using a set of 4,729,872 SNPs with a minimum imputation accuracy score of ≥0.9 and call rate of ≥0.99 in both the PsA‐BSTOP GWAS and the UK Biobank GWAS ICD‐10 data sets in which the PsA‐BSTOP data set was used as the training data set and the UK Biobank ICD‐10 data set was used for external validation (see Supplementary Figure 4B, available at http://onlinelibrary.wiley.com/doi/10.1002/art.42154). We utilized a lasso‐penalized linear regression model using all post–quality control imputed SNPs where the penalty (L1) was tuned with 10 repetitions of 10‐fold cross‐validation implemented in the SparSNP software package (20). The best model was selected based on the maximal area under the curve (AUC) and classification and calibration were evaluated in the validation data set.

## RESULTS

### Heritability estimates

We calculated the SNP‐based heritability (h^2^_SNP_) of PsA in the PsA‐BSTOP GWAS data set of 3,609 patients and 9,192 controls. The estimated heritability of PsA in the full data set was h^2^_SNP_ = 0.63 (SD 0.04), while in analyses using non‐MHC SNPs, the estimated heritability was h^2^_SNP_ = 0.61 (SD 0.04).

In analyses in which the disease prevalence was specified to be 1% (in comparison to previous prevalence estimates [7]), the estimated heritability of PsA was h^2^_SNP_ = 0.43 (SD 0.03), while the heritability of PsA in analyses using non‐MHC SNPs was h^2^_SNP_ = 0.41 (SD 0.03).

The heritability of PsC in a population of 2,085 patients and 9,192 controls was estimated to be h^2^_SNP_ = 0.61 (SD 0.05), while in analyses using non‐MHC SNPs, the estimated heritability of PsC was h^2^_SNP_ = 0.59 (SD 0.05). With a disease prevalence of 1%, the estimated heritability of PsC was found to be h^2^_SNP_ = 0.56 (SD 0.04), while the heritability of PsC in analyses using non‐MHC SNPs was h^2^_SNP_ = 0.54 (SD 0.05).

### Association testing and meta‐analysis

**Figure 1:**
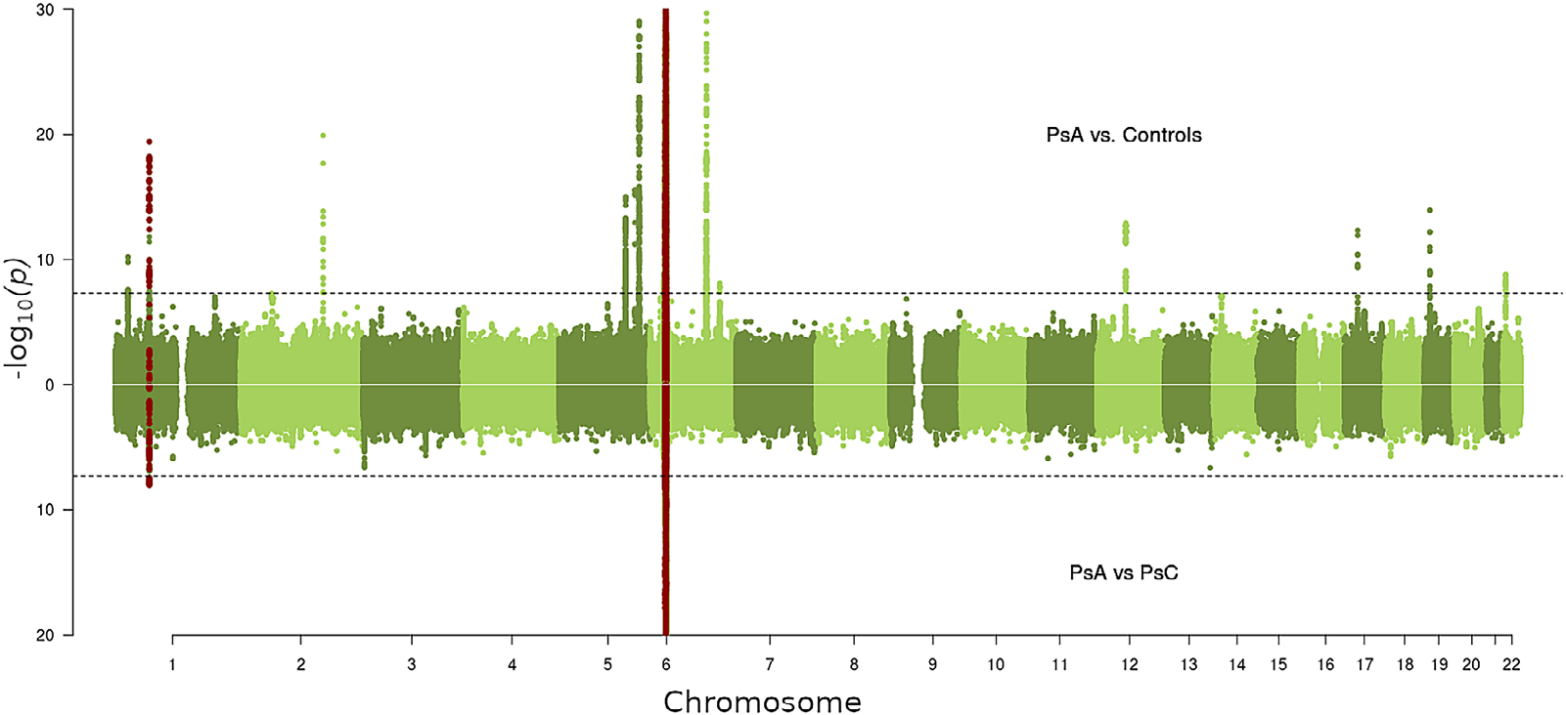
Manhattan plots showing the *P* values of genome‐wide significance from the meta‐analysis of summary statistics obtained from psoriatic arthritis (PsA) patients compared to population controls (top), and PsA patients compared to cutaneous‐only psoriasis (PsC) patients (bottom). The genome‐wide significance threshold was set at *P* = 5 × 10^−8^ and is indicated by the dashed lines. Each dot represents a single‐nucleotide polymorphism (SNP). Red dots indicate the most significant SNPs in both data sets.

We performed a meta‐analysis of GWAS summary statistics from a total of 5,065 PsA patients and 21,286 controls for a maximum of 8,558,403 SNPs using data from the PsA‐BSTOP, the UK Biobank, and the PsA Immunochip data sets (see Supplementary Figure 3A, available at http://onlinelibrary.wiley.com/doi/10.1002/art.42154). The genomic control inflation factor λ_gc_ (λ_gc1000_) for the PsA‐BSTOP GWAS data set was estimated to be 1.1 (1.01), indicating minimal residual population stratification based on inflation of test statistics. We identified 16 non‐MHC loci with genome‐wide significance for the development of PsA (*P* = 5 × 10^−8^), 15 of which have previously been reported as significant in PsA and one which is novel (Table 1 and Figure 1). This novel genome‐wide association represents an association with the intergenic SNP rs5754467 (*P* = 1.61 × 10^−9^) on chromosome 22q11 in close proximity to the gene *UBE2L3*. We also found that the 2 previously reported PsA‐specific susceptibility loci *PTPN22* (rs2476601; *P* = 6.03 × 10^−7^) and chr5q31 (rs715285; *P* = 2.86 × 10^−11^) had genome‐wide significance for the development of PsA.

**Table 1:** Non‐MHC loci with genome‐wide significance in the development of PsA identified through a meta‐analysis of GWAS summary statistics from PsA patients and controls*

SNP	Chromosome	Base position	Notable genes	Risk/non‐risk allele	RAF	*P*	OR (95% CI)	*P* by Cochran's Q test	I^2^
rs33980500	6	111913262	*TRAF3IP2*	T/C	0.07	1.14 × 10^−36^	1.66 (1.54–1.8)	0.48	0
rs62377586	5	158766022	*IL12B*	G/A	0.67	8.17 × 10^−35^	1.36 (1.3–1.43)	0.52	0
rs2111485	2	163110536	*IFIH1*	G/A	0.61	1.24 × 10^−20^	1.25 (1.19–1.31)	0.80	0
rs12044149	1	67600686	*IL23R*	T/G	0.26	3.84 × 10^−20^	1.27 (1.2–1.33)	0.27	0.23
rs76956521	5	150464641	*TNIP1*	C/A	0.05	2.65 × 10^−16^	1.49 (1.36–1.64)	0.82	0
rs848	5	131996500	*IL13*	C/A	0.82	9.49 × 10^−16^	1.28 (1.21–1.36)	0.65	0
rs34536443	19	10463118	*TYK2*	G/C	0.95	1.16 × 10^−14^	1.71 (1.49–1.96)	0.70	0
rs17622208	5	131717050	*SLC22A5*	A/G	0.48	5.73 × 10^−14^	1.19 (1.14–1.24)	0.12	0.53
rs2020854	12	56743367	*STAT2*	T/C	0.93	1.26 × 10^−13^	1.43 (1.3–1.57)	0.01	0.78
rs3794767	17	26124605	*NOS2*	C/T	0.64	4.73 × 10^−13^	1.19 (1.14–1.25)	0.83	0
rs13203885	6	111995127	*FYN*	C/T	0.12	1.55 × 10^−11^	1.26 (1.18–1.35)	0.74	0
rs1395621	1	25270572	*RUNX3*	C/T	0.48	6.48 × 10^−11^	1.17 (1.12–1.23)	0.65	0
rs5754467†	22	21985094	*CCDC116*	G/A	0.19	1.61 × 10^−9^	1.19 (1.13–1.27)	0.85	0
rs610604	6	138199417	*TNFAIP3*	G/T	0.32	7.76 × 10^−9^	1.15 (1.1–1.21)	0.14	0.50

* Inconsistency metrics (I^2^) describing the percentage of variation across studies due to heterogeneity were assessed for significance by Cochran's Q heterogeneity test. The threshold for genome‐wide significance was *P* = 5 × 10^−8^. MHC = major histocompatibility complex; GWAS = genome‐wide association study; SNP = single‐nucleotide polymorphism; RAF = risk allele frequency; OR = odds ratio; 95% CI = 95% confidence interval.

† Novel locus not previously identified as significant in the development of psoriatic arthritis (PsA).

Next, we performed a meta‐analysis of summary statistics for the comparison of PsA to PsC (PsA‐BSTOP and UK Biobank data) to identify PsA‐specific susceptibility loci using a population consisting of 4,340 PsA patients and 6,431 PsC patients (see Supplementary Figure 3B, available at http://onlinelibrary.wiley.com/doi/10.1002/art.42154). We identified significant genome‐wide association in 2 loci previously reported to be associated with the development of PsA, namely the MHC region (rs1050414; *P* = 8.49 × 10^−59^) and the *IL23R* gene (rs72676069; *P* = 9.94 × 10^−9^). No other regions reached genome‐wide significance. However, 4 loci demonstrated evidence of significant association in both data sets, with an overall *P* value in the meta‐analysis of *P* < 5 × 10^−6^ (Table 2), giving us confidence in the existence of additional PsA‐specific loci.

**Table 2:** Loci showing the most significant association with PsA or PsC from the PsA‐STOP, UK Biobank, and meta‐analysis data sets*

	SNPs			
	rs17194140	rs11665266	rs76800961	rs306281
Chromosome	3	18	14	7
Base position	2198673	10441470	85656555	154785362
Notable genes	*CNTN4*	None	None	*PAXIP1*
Risk/non‐risk allele	T/C	A/G	A/C	G/A
*P* for association, PsA‐BSTOP data set	2.75 × 10^–5^	0.00304	3.33 × 10^–5^	1.81 × 10^–4^
*P* for association, UK Biobank data set	2.62 × 10^–3^	6.35 × 10^–5^	2.30 × 10^–2^	6.92 × 10^–3^
*P* for association, meta‐analysis data set	2.51 × 10^–7^	1.96 × 10^–6^	2.61 × 10^–6^	3.97 × 10^–6^
OR (95% CI)	1.2 (1.12–1.29)	1.34 (1.19–1.51)	1.39 (1.21–1.59)	1.17 (1.09–1.24)
*P* by Cochran's Q test	0.97	0.15	0.60	0.95
I^2^	0.00	0.53	0.00	0.00

* The overall *P* value for the meta‐analysis was *P* = 5 × 10^−6^. Inconsistency metrics (I^2^) describing the percentage of variation across studies due to heterogeneity were assessed for significance by Cochran's Q heterogeneity test. See Table 1 for definitions.

### Gene and pathway prioritization

We utilized the recently described Pi bioinformatics pipeline to identify key genes and pathways in the development of PsA (19). Using summary statistics from the meta‐analyses described above for PsA patients versus controls, we found that the most highly ranked gene with regard to PsA susceptibility based on the Pi was *ICAM1*, which has a role in epithelial cell adhesion (see Supplementary Table 3, available at http://onlinelibrary.wiley.com/doi/10.1002/art.42154). In addition, several genes involved in IFN regulation were highly ranked (*IRF1*, *IRF5* and *IRF7*). Other highly ranked genes included *UBA52*, *CNPY2*, *STAT2*, and *TYK2*. Using the top 1% of ranked genes, we found significant enrichment in IFN and interleukin signaling pathways (see Supplementary Table 4, available at http://onlinelibrary.wiley.com/doi/10.1002/art.42154). These pathways were not found to be enriched when using summary statistics from PsA patients compared to those from PsC patients, suggesting that these pathways are primarily involved in the pathogenesis of psoriasis (see Supplementary Tables 5 and 6, available at http://onlinelibrary.wiley.com/doi/10.1002/art.42154). Pathways found to be enriched in the comparison of PsA to PsC included multiple pathways for NF‐κB signaling (adjusted *P* = 1.4 × 10^−45^) and Wnt signaling (adjusted *P* = 9.5 × 10^−58^), which provides compelling evidence that these pathways are potentially involved in the development of PsA.

### Risk prediction

**Figure 2:**
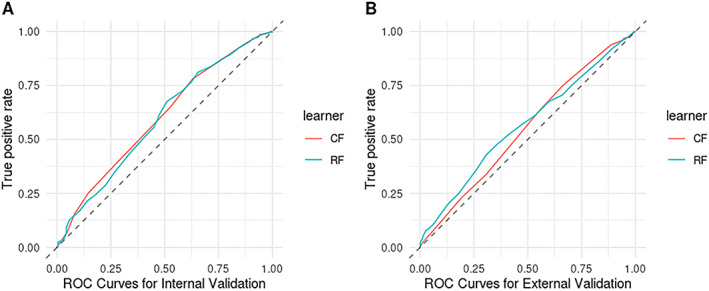
Receiver operating characteristic (ROC) curves showing the sensitivity and specificity of the random forest (RF) and the conditional inference forest (CF) machine learning algorithms in discriminating between psoriatic arthritis (PsA) and cutaneous‐only psoriasis (PsC). Both the RF and CF models showed modest performance across the PsA‐Biomarkers of Systemic Treatment Outcomes in Psoriasis (PsA‐BSTOP) study data set (**A**) and the UK Biobank International Statistical Classification of Diseases and Related Health Problems, Tenth Revision data set (**B**). The Concordance statistic for each model was <0.6 by external validation.

We assessed the ability of the Michigan classification pipeline to discriminate PsA from PsC in our available data sets (see Supplementary Figure 4A). The 2 reported statistical approaches (the random forest model and the conditional inference forest model) performed poorly across both the training data set (PsA‐BSTOP) and the validation data set (UK Biobank ICD‐10), with C statistics of <0.6 by external validation (Figure 2). Each model was characterized by high sensitivity but low specificity, indicating a high rate of false positives (see Supplementary Table 7, available at http://onlinelibrary.wiley.com/doi/10.1002/art.42154). In addition, calibration and clinical utility were found to be poor for both the random forest model and the conditional inference forest model (see Supplementary Figures 5 and 6). The best performing model based on accuracy of discrimination in the validation data set was the random forest model, where the C statistic was found to be 0.61 (95% confidence interval [95% CI] 0.56–0.76) in internal validation, but which dropped considerably to 0.57 (95% CI 0.54–0.61) in external validation. The overall performance of the random forest model as measured by the Brier score was similar for internal and external validation, with Brier scores of 0.22 and 0.30, respectively, suggesting poor agreement in both data sets. The random forest model was also found to be poorly calibrated, with a calibration‐in‐the‐large (CITL) score of 0.27 (95% CI –0.13, 0.69) in internal validation and a noticeably worse CITL score of 1.2 (95% CI 0.92–1.51) in external validation.

Finally, we used the PsA‐BSTOP GWAS data set to develop a PsA risk prediction model using a set of 4,729,872 SNPs and lasso‐penalized linear regression (see Supplementary Figure 4B, available at http://onlinelibrary.wiley.com/doi/10.1002/art.42154). The best model achieved an AUC of 0.66 when assessed using 10‐fold cross‐validation and consisted of 118 SNPs, 34 of which mapped to the MHC (see Supplementary Figure 7). The SNP weights, *P* values, and model intercept are reported in Supplementary Table 8, available at http://onlinelibrary.wiley.com/doi/10.1002/art.42154. Independent validation of this model in the UK Biobank GWAS data set demonstrated an AUC of 0.57. The optimal prediction cutoff value to maximize the true‐positive rate and minimize the false‐positive rate was 0.3, which resulted in a sensitivity of 0.53 and a specificity of 0.58. Calibration of this model was found to be poor, with a CITL score of –2.16 (95% CI –2.31, –2.01) (see Supplementary Figure 8), suggesting a general overestimation of risk, and a calibration slope of 1.41 (95% CI 0.95–1.86), suggesting that the predictions were too moderate and showing limited variation in the predicted probabilities.

## DISCUSSION

Using a large integrated data set of PsA patients, PsC patients, and controls, we have been able to provide accurate heritability estimates, identify a novel susceptibility locus, explore key biologic pathways associated with the development of PsA, and explore the utility of prediction models for classifying PsA risk. While the individual SNP analysis showed large overlap between PsC and PsA, pathway analysis revealed important differences, including enrichment of PsA‐significant SNPs found in key pathways such as NF‐κB and Wnt signaling.

The SNP‐based heritability estimates reported herein support recent findings by Li et al (7) and show comparable heritability of PsA and PsC. Our results do not support the previous family and population estimates that suggest a substantially larger heritable component for PsA above that of psoriasis alone (4, 5, 6). Seventeen genome‐wide associations were identified, including rs5754467 (*P* = 1.61 × 10^−9^) which maps to chromosome 22q11 and is near the genes *UBE2L3, YDJC*, and *CCDC116*. This SNP has not been previously reported in the setting of PsA, but is highly correlated (r^2^ >0.8 based on SNP data from a northern European population) with a previously identified psoriasis SNP. This correlation does not represent a PsA‐specific genetic effect (12), but further supports the genetic similarity of psoriasis in both patients with PsA and patients with PsC.

We used the Pi pipeline to identify key genes and pathways involved in PsA susceptibility. In analyses of PsA patients compared to controls, we replicated the previously reported findings of prioritized genes (*ICAM1, IRF1, STAT2*, and *TYK2*) and target pathways (IFN and interleukin signaling) for psoriasis, further supporting the notion that psoriasis in patients with PsA is genetically and biologically similar to psoriasis in patients with PsC. Interestingly, these pathways for PsA development were reported previously in a study applying the Pi pipeline to a set of 59 SNPs associated with PsA (21). However, of greatest interest is the prioritized target pathways that differ between PsA and PsC, which provide insight into the PsA‐specific processes whereby we find enrichment in multiple NF‐κB signaling annotations and the Wnt signaling pathway.

The Wnt signaling pathway plays a key role in bone formation in normal development and in abnormal bone formation in diseases such as axial spondyloarthritis and osteoarthritis. The Wnt signaling pathway may also be of particular interest in the setting of PsA, where bone formation in peripheral joints is included in the CASPAR criteria for the classification of PsA. Blocking of Dkk‐1 (an inhibitor of Wnt signaling) in mice has been shown to halt progressive and erosive joint destruction in inflammatory arthritis by encouraging bone formation (22). Interestingly, a previous study on PsA demonstrated that PsA patients had lower levels of Dkk‐1 compared to healthy controls, and treatment with secukinumab increased these levels over a period of 6 months to normal Dkk‐1 serum levels (23). In contrast, another study reported no significant difference in levels of Dkk‐1 in patients with PsA without radiographic axial disease compared to healthy controls (24). Therefore, further work is required to understand the role of Wnt signaling in PsA.

A previous study by Aterido et al investigated pathways associated with PsA susceptibility and reported significant association with the glycosaminoglycan metabolism pathway (Reactome R‐HSA‐1630316) (25). However, no association between PsA susceptibility and the glycosaminoglycan metabolism pathway was observed in our data, as none of the highly prioritized genes overlapped with genes in this pathway annotation. These differing results could be attributed to differences in the methods used for mapping SNPs to genes, as in the study by Aterido et al SNPs were assigned to genes based solely on proximity. It is now well recognized that causal genes are not always those that are closest to the GWAS hit, and the causal SNP may exert its regulatory effect on distant genes. The Pi pipeline addresses this limitation by including gene expression data and chromatin confirmation data in order to capture evidence for SNP–gene physical interactions in addition to proximity information (19). Aterido et al also reported that the SNP rs10865331 at the *B3GNT2* locus was associated with the risk of developing PsA but not PsC (*P* = 0.029). While we found significant association with this SNP when comparing PsA patients to controls (*P* = 2.05 × 10^−7^), we found no evidence that this association is PsA‐specific based on our stratified analyses comparing PsA to PsC using PsA‐BSTOP data (*P* = 0.41) or on the larger meta‐analysis using UK Biobank and Immunochip data (P = 0.31).

Our prediction models showed only modest ability to discriminate PsA from PsC in the available data sets, which was consistent with the findings of a recently published study by Smith et al (26). For the first approach, we attempted to reproduce a previously published classification pipeline by following the description and published model parameters, which allowed us to reproduce the workflows (13). Following the author recommendations, we used the 2 sets of genetic features that were selected using a well‐phenotyped cohort and estimated the model parameters in our data to capture cohort‐specific effects and optimize performance. Second, we attempted to develop a model for our existing data sets through external validation. However, neither of these approaches achieved satisfactory discrimination either with internal or external validation. Given that the predictive performance of a model using the same data on which it was developed (often referred to as apparent performance) will tend to give an optimistic estimate of the model's performance, it is not uncommon for a prediction model to achieve lower performance results when applied to an external population.

The lack of discrimination observed in our data sets could be due to the differences in demographic and clinical characteristics of participants in our data sets compared to those of the participants in the original study. PsA is clinically a heterogeneous disease and differing proportions of patients with oligoarticular or polyarticular arthritis mutilans and axial disease (each with potentially differing genetic risk factors) could have contributed to the decreased performance of the model. This potential issue was recognized by the authors of the original study and, although we followed the author recommendations by modeling the effects of these markers in our data to learn these cohort‐specific parameters, the overall classification performance remained low (13).

An important limitation of our study was the potential impact of poor phenotype specificity in the PsC cohorts where the existence of undiagnosed PsA could have confounded the performance of any classification models. Although the participants in the BSTOP study were not screened for the absence of PsA by a rheumatologist, they were routinely followed‐up with an average of 8 consultations and had a psoriasis disease duration of 27 years without a recorded diagnosis of PsA. Additionally, restricting analyses to a subgroup of PsC patients with psoriasis for a duration of ≥10 (to minimize the risk of undiagnosed PsA) did not improve the performance of the predictive model (data not shown). However, given the extent of undiagnosed PsA in dermatology clinics, we cannot exclude the possibility of undiagnosed PsA in this group, which would have impacted both feature selection and model performance (27). Furthermore, given that patients in the PsC group were treated with biologic drugs that are also effective in the treatment of PsA, it is possible that PsA development was prevented in susceptible individuals, thus limiting the power of the models to discriminate between groups (28).

In conclusion, predicting the risk of PsA development in patients with psoriasis remains an important research question, and external validation in addition to statistical validation is an important step in the clinical translation of PsA prediction models, as external validation tests the transportability of models to plausibly related populations (29). While polygenic risk scores capture the heritable component of disease susceptibility, they fail to capture the more dynamic risk factors that can modulate susceptibility, such as environmental and lifestyle risk factors. In addition, studies have shown that genetic risk factors can be independent of known clinical risk factors (30). This suggests that future research on PsA susceptibility in patients with psoriasis should move toward combining clinical data and genetics from data collected longitudinally, using a prospective study design in patients with clinically well‐defined PsC before treatment with biologic drugs, to create an integrated risk score. Therefore, these future efforts should also investigate the integration of more dynamic biomarkers, such as the host microbiome and immunophenotyping, into the development of PsA risk prediction models.

## AUTHOR CONTRIBUTIONS

All authors were involved in drafting the article or revising it critically for important intellectual content, and all authors approved the final version to be published. Dr. Barton had full access to all of the data in the study and takes responsibility for the integrity of the data and the accuracy of the data analysis.

### Study conception and design

Soomro, Stadler, Dand, Barker, Smith, Bowes, Barton.

### Acquisition of data

Dand, Jadon, Duckworth, Ho, Marzo‐Ortega, Helliwell, Ryan, Kane, Korendowych, Simpson, Packham, McManus, Gabay, Lamacchia, Nissen, Brown, Verstappen, Barker, Smith, FitzGerald, McHugh, Warren, Bowes, Barton.

### Analysis and interpretation of data

Soomro, Stadler, Dand, Bluett, Jalali‐najafabadi, Duckworth, Van Staa, Barker, Smith, Bowes, Barton.

## ADDITIONAL DISCLOSURES

Author Ryan is an employee of Genuity Science Dublin. Author Brown is an employee of Genomics England.

## Supporting information


Disclosure Form

**Appendix S1** Supporting Information

**Supplementary table 1** | summary of clinical phenotype data for available psoriatic arthritis patients
**Supplementary table 2** | two sets of genetic features used witin the Michigan classification pipeline
**Supplementary table 3** | Priority Index gene prioritisation of PsA compared to population controls (top 100)
**Supplementary table 4** | Priority Index pathway prioritisation for PsA compared to population controls
**Supplementary table 5** | Priority Index gene prioritisation of PsA compared to PsC (top 100)
**Supplementary table 6** | Priority Index pathway prioritisation for PsA compared to PsC
**Supplementary table 7** | Classification performance metrics for random forest and conditional random forest in internal validation (IV) using the PsA‐BSTOP GWAS and external validation (EV) using the UK Biobank ICD10 dataset
**Supplementary table 8** | SNP weight for best model from SparSNP cross‐validation (intercept = 0.335167696577)

## Data Availability

Summary statistics of the GWAS analyzed in the current study are available through the National Human Genome Research Institute‐European Bioinformatics Institute GWAS Catalog at https://www.ebi.ac.uk/gwas/downloads/summary-statistics. Control population data were obtained from the UK Household Longitudinal Study. Information on how to access the data can be found on the Understanding Society website at https://www.understandingsociety.ac.uk/.
