# Supplementary material for: Comparative Genetic Analysis of Psoriatic Arthritis and Psoriasis for the Discovery of Genetic Risk Factors and Risk Prediction Modeling
Source: Arthritis Rheumatol. 2022 Aug 4;74(9):1535–43. doi: 10.1002/art.42154 (PMC9539852; doi:10.1002/art.42154)
Supplement: Supplementary file 1 — Disclosure Form [file ART-74-1535-s002.pdf]

31/01/2022

ICMJE DISCLOSURE FORM

Date:____
Your Name:___Anne Barton____
Manuscript Title:_ Comparative genetic analysis of psoriatic arthritis and psoriasis for the discovery of genetic risk factors and risk prediction modelling____
Manuscript number (if known):_ ar-21-1232____

In the interest of transparency, we ask you to disclose all relationships/activities/interests listed below that are related to the content of your manuscript. "Related" means any relation with for-profit or not-for-profit third parties whose interests may be affected by the content of the manuscript. Disclosure represents a commitment to transparency and does not necessarily indicate a bias. If you are in doubt about whether to list a relationship/activity/interest, it is preferable that you do so.

The following questions apply to the author's relationships/activities/interests as they relate to the current manuscript only.

The author's relationships/activities/interests should be defined broadly. For example, if your manuscript pertains to the epidemiology of hypertension, you should declare all relationships with manufacturers of antihypertensive medication, even if that medication is not mentioned in the manuscript.

In item #1 below, report all support for the work reported in this manuscript without time limit. For all other items, the time frame for disclosure is the past 36 months.

		**Name all entities with whom you have this relationship or indicate none (add rows as needed)**	**Specifications/Comments (e.g., if payments were made to you or to your institution)**
**Time frame: Since the initial planning of the work**			
1	All support for the present manuscript (e.g., funding, provision of study materials, medical writing, article processing charges, etc.) **No time limit for this item.**	Versus Arthritis	Grant funding to Institution
		NIHR	Grant funding to Institution
**Time frame: past 36 months**			
2	Grants or contracts from any entity (if not indicated in item #1 above).	__X__None	
3	Royalties or licenses	__X__None	
4	Consulting fees	__X__None	

5	Payment or honoraria for lectures, presentations, speakers bureaus, manuscript writing or educational events	__X__None	
6	Payment for expert testimony	__X__None	
7	Support for attending meetings and/or travel	__X__None	
8	Patents planned, issued or pending	__X__None	
9	Participation on a Data Safety Monitoring Board or Advisory Board	__X__None	
10	Leadership or fiduciary role in other board, society, committee or advocacy group, paid or unpaid	__X__None	
11	Stock or stock options	__X__None	
12	Receipt of equipment, materials, drugs, medical writing, gifts or other services	__X__None	
13	Other financial or non-financial interests	__X__None	

Please place an "X" next to the following statement to indicate your agreement:

X_ I certify that I have answered every question and have not altered the wording of any of the questions on this form.

ICMJE DISCLOSURE FORM

Date:___25/02/2022____
Your Name:___Anthony Ryan____
Manuscript Title:_ Comparative genetic analysis of psoriatic arthritis and psoriasis for the discovery of genetic risk factors and risk prediction modelling____
Manuscript number (if known):_ ar-21-1232____

In the interest of transparency, we ask you to disclose all relationships/activities/interests listed below that are related to the content of your manuscript. "Related" means any relation with for-profit or not-for-profit third parties whose interests may be affected by the content of the manuscript. Disclosure represents a commitment to transparency and does not necessarily indicate a bias. If you are in doubt about whether to list a relationship/activity/interest, it is preferable that you do so.

The following questions apply to the author's relationships/activities/interests as they relate to the current manuscript only.

The author's relationships/activities/interests should be defined broadly. For example, if your manuscript pertains to the epidemiology of hypertension, you should declare all relationships with manufacturers of antihypertensive medication, even if that medication is not mentioned in the manuscript.

In item #1 below, report all support for the work reported in this manuscript without time limit. For all other items, the time frame for disclosure is the past 36 months.

		**Name all entities with whom you have this relationship or indicate none (add rows as needed)**	**Specifications/Comments (e.g., if payments were made to you or to your institution)**
		**Time frame: Since the initial planning of the work**	
1	All support for the present manuscript (e.g., funding, provision of study materials, medical writing, article processing charges, etc.) **No time limit for this item.**	__X__None	
		**Time frame: past 36 months**	
2	Grants or contracts from any entity (if not indicated in item #1 above).	__X__None	
3	Royalties or licenses	_X___None	
4	Consulting fees	__X__None	

5	Payment or honoraria for lectures, presentations, speakers bureaus, manuscript writing or educational events	__X__None	
6	Payment for expert testimony	__X__None	
7	Support for attending meetings and/or travel	__X__None	
8	Patents planned, issued or pending	__X__None	
9	Participation on a Data Safety Monitoring Board or Advisory Board	__X__None	
10	Leadership or fiduciary role in other board, society, committee or advocacy group, paid or unpaid	__X__None	
11	Stock or stock options	__X__None	
12	Receipt of equipment, materials, drugs, medical writing, gifts or other services	__X__None	
13	Other financial or non-financial interests	__X__None	

Please place an "X" next to the following statement to indicate your agreement:

_X_ I certify that I have answered every question and have not altered the wording of any of the questions on this form.

ICMJE DISCLOSURE FORM

Date:______03/03/2022____
Your Name:______Cem Gaby____
Manuscript Title:_ Comparative genetic analysis of psoriatic arthritis and psoriasis for the discovery of genetic risk factors and risk prediction modelling____
Manuscript number (if known):_ ar-21-1232____

In the interest of transparency, we ask you to disclose all relationships/activities/interests listed below that are related to the content of your manuscript. "Related" means any relation with for-profit or not-for-profit third parties whose interests may be affected by the content of the manuscript. Disclosure represents a commitment to transparency and does not necessarily indicate a bias. If you are in doubt about whether to list a relationship/activity/interest, it is preferable that you do so.

The following questions apply to the author's relationships/activities/interests as they relate to the current manuscript only.

The author's relationships/activities/interests should be defined broadly. For example, if your manuscript pertains to the epidemiology of hypertension, you should declare all relationships with manufacturers of antihypertensive medication, even if that medication is not mentioned in the manuscript.

In item #1 below, report all support for the work reported in this manuscript without time limit. For all other items, the time frame for disclosure is the past 36 months.

		**Name all entities with whom you have this relationship or indicate none (add rows as needed)**	**Specifications/Comments (e.g., if payments were made to you or to your institution)**
		**Time frame: Since the initial planning of the work**	
1	All support for the present manuscript (e.g., funding, provision of study materials, medical writing, article processing charges, etc.) **No time limit for this item.**	__X__None	
		**Time frame: past 36 months**	
2	Grants or contracts from any entity (if not indicated in item #1 above).	__X__None	
3	Royalties or licenses	__X__None	
4	Consulting fees	__X__None	

5	Payment or honoraria for lectures, presentations, speakers bureaus, manuscript writing or educational events	__X__None	
6	Payment for expert testimony	__X__None	
7	Support for attending meetings and/or travel	__X__None	
8	Patents planned, issued or pending	__X__None	
9	Participation on a Data Safety Monitoring Board or Advisory Board	__X__None	
10	Leadership or fiduciary role in other board, society, committee or advocacy group, paid or unpaid	__X__None	
11	Stock or stock options	__X__None	
12	Receipt of equipment, materials, drugs, medical writing, gifts or other services	__X__None	
13	Other financial or non-financial interests	__X__None	

Please place an "X" next to the following statement to indicate your agreement:

__X_ I certify that I have answered every question and have not altered the wording of any of the questions on this form.

ICMJE DISCLOSURE FORM

Date:_______03/03/2022____
Your Name:_______Celine Lamacchia____
Manuscript Title:_ Comparative genetic analysis of psoriatic arthritis and psoriasis for the discovery of genetic risk factors and risk prediction modelling____
Manuscript number (if known):_ ar-21-1232____

In the interest of transparency, we ask you to disclose all relationships/activities/interests listed below that are related to the content of your manuscript. "Related" means any relation with for-profit or not-for-profit third parties whose interests may be affected by the content of the manuscript. Disclosure represents a commitment to transparency and does not necessarily indicate a bias. If you are in doubt about whether to list a relationship/activity/interest, it is preferable that you do so.

The following questions apply to the author's relationships/activities/interests as they relate to the current manuscript only.

The author's relationships/activities/interests should be defined broadly. For example, if your manuscript pertains to the epidemiology of hypertension, you should declare all relationships with manufacturers of antihypertensive medication, even if that medication is not mentioned in the manuscript.

In item #1 below, report all support for the work reported in this manuscript without time limit. For all other items, the time frame for disclosure is the past 36 months.

		**Name all entities with whom you have this relationship or indicate none (add rows as needed)**	**Specifications/Comments (e.g., if payments were made to you or to your institution)**
		**Time frame: Since the initial planning of the work**	
1	All support for the present manuscript (e.g., funding, provision of study materials, medical writing, article processing charges, etc.) **No time limit for this item.**	__X__None	
		**Time frame: past 36 months**	
2	Grants or contracts from any entity (if not indicated in item #1 above).	__X__None	
3	Royalties or licenses	__X__None	
4	Consulting fees	__X__None	

5	Payment or honoraria for lectures, presentations, speakers bureaus, manuscript writing or educational events	__X__None	
6	Payment for expert testimony	__X__None	
7	Support for attending meetings and/or travel	__X__None	
8	Patents planned, issued or pending	__X__None	
9	Participation on a Data Safety Monitoring Board or Advisory Board	__X__None	
10	Leadership or fiduciary role in other board, society, committee or advocacy group, paid or unpaid	__X__None	
11	Stock or stock options	__X__None	
12	Receipt of equipment, materials, drugs, medical writing, gifts or other services	__X__None	
13	Other financial or non-financial interests	__X__None	

Please place an “X” next to the following statement to indicate your agreement:

_X__ I certify that I have answered every question and have not altered the wording of any of the questions on this form.

ICMJE DISCLOSURE FORM

Date:_25/2/2022____
Your Name:______Catherine Smith____
Manuscript Title:_ Comparative genetic analysis of psoriatic arthritis and psoriasis for the discovery of genetic risk factors and risk prediction modelling____
Manuscript number (if known):_ ar-21-1232____

In the interest of transparency, we ask you to disclose all relationships/activities/interests listed below that are related to the content of your manuscript. "Related" means any relation with for-profit or not-for-profit third parties whose interests may be affected by the content of the manuscript. Disclosure represents a commitment to transparency and does not necessarily indicate a bias. If you are in doubt about whether to list a relationship/activity/interest, it is preferable that you do so.

The following questions apply to the author's relationships/activities/interests as they relate to the current manuscript only.

The author's relationships/activities/interests should be defined broadly. For example, if your manuscript pertains to the epidemiology of hypertension, you should declare all relationships with manufacturers of antihypertensive medication, even if that medication is not mentioned in the manuscript.

In item #1 below, report all support for the work reported in this manuscript without time limit. For all other items, the time frame for disclosure is the past 36 months.

		**Name all entities with whom you have this relationship or indicate none (add rows as needed)**	**Specifications/Comments (e.g., if payments were made to you or to your institution)**
		**Time frame: Since the initial planning of the work**	
1	All support for the present manuscript (e.g., funding, provision of study materials, medical writing, article processing charges, etc.) **No time limit for this item.**	__Psoriasis Association	Grant for accrual of the bioresource BSTOP (contributed data to the analysis)
		Guys and St Thomas' NIHR Biomedical Research Centre	Core funding to support bioresourcing including BSTOP (contributed data to the analysis)
		Medical Research Council Psoriasis Stratification to Optimise Relevant Therapy (PSORT)	Grant contributed to generation of data used in this analysis. PSORT has a number of industry partners who manufacturer therapeutics for use in psoriasis and psoriatic arthritis see PSORT.org.uk for fulling listing
		**Time frame: past 36 months**	

2	Grants or contracts from any entity (if not indicated in item #1 above).	MRC-Phd studentship co-funded by Boeringher Ingleheim	BI manufacture therapeutics for use in psoriasis and psA
		IMI (Horizon 2020) funded European consortium with multiple industry partners grants	Industry partners that are part of this consortium manufacture therapeutics for psoriasis and psoriatic arthritis see biomap-imi.eu for details
3	Royalties or licenses	__X__None	
4	Consulting fees	__X__None	
5	Payment or honoraria for lectures, presentations, speakers bureaus, manuscript writing or educational events	__X__None	
6	Payment for expert testimony	__X__None	
7	Support for attending meetings and/or travel	__X__None	
8	Patents planned, issued or pending	__X__None	
9	Participation on a Data Safety Monitoring Board or Advisory Board	__X__None	
10	Leadership or fiduciary role in other board, society, committee or advocacy group, paid or unpaid	__X__None	
11	Stock or stock options	__X__None	
12	Receipt of equipment, materials, drugs, medical writing, gifts or other services	__X__None	
13	Other financial or non-financial interests	___Participant in UK and European guidelines for management of psoriasis /psoriatic arthritis (chair/member of committee)	No financial interest (personal or non personal). Opinion leader.

Please place an "X" next to the following statement to indicate your agreement:

__x_ I certify that I have answered every question and have not altered the wording of any of the questions on this form.

ICMJE DISCLOSURE FORM

Date: 11 Feb 2022
Your Name: Dr Deepak Jadon
Manuscript Title:_ Comparative genetic analysis of psoriatic arthritis and psoriasis for the discovery of genetic risk factors and risk prediction modelling
Manuscript number (if known):_ ar-21-1232

In the interest of transparency, we ask you to disclose all relationships/activities/interests listed below that are related to the content of your manuscript. "Related" means any relation with for-profit or not-for-profit third parties whose interests may be affected by the content of the manuscript. Disclosure represents a commitment to transparency and does not necessarily indicate a bias. If you are in doubt about whether to list a relationship/activity/interest, it is preferable that you do so.

The following questions apply to the author's relationships/activities/interests as they relate to the current manuscript only.

The author's relationships/activities/interests should be defined broadly. For example, if your manuscript pertains to the epidemiology of hypertension, you should declare all relationships with manufacturers of antihypertensive medication, even if that medication is not mentioned in the manuscript.

In item #1 below, report all support for the work reported in this manuscript without time limit. For all other items, the time frame for disclosure is the past 36 months.

		Name all entities with whom you have this relationship or indicate none (add rows as needed)	Specifications/Comments (e.g., if payments were made to you or to your institution)
**Time frame: Since the initial planning of the work**			
1	All support for the present manuscript (e.g., funding, provision of study materials, medical writing, article processing charges, etc.) **No time limit for this item.**	__X__None	
**Time frame: past 36 months**			
2	Grants or contracts from any entity (if not indicated in item #1 above).	__X__None	
3	Royalties or licenses	__X__None	
4	Consulting fees	__X__None	

5	Payment or honoraria for lectures, presentations, speakers bureaus, manuscript writing or educational events	__X__None	
6	Payment for expert testimony	_X___None	
7	Support for attending meetings and/or travel	__X__None	
8	Patents planned, issued or pending	__X__None	
9	Participation on a Data Safety Monitoring Board or Advisory Board	__X__None	
10	Leadership or fiduciary role in other board, society, committee or advocacy group, paid or unpaid	_X___None	
11	Stock or stock options	__X__None	
12	Receipt of equipment, materials, drugs, medical writing, gifts or other services	__X__None	
13	Other financial or non-financial interests	_X___None	

Please place an "X" next to the following statement to indicate your agreement:

X I certify that I have answered every question and have not altered the wording of any of the questions on this form.

ICMJE DISCLOSURE FORM

Date:_________25/2/2022____
Your Name:____David Kane____
Manuscript Title:_ Comparative genetic analysis of psoriatic arthritis and psoriasis for the discovery of genetic risk factors and risk prediction modelling____
Manuscript number (if known):_ ar-21-1232____

In the interest of transparency, we ask you to disclose all relationships/activities/interests listed below that are related to the content of your manuscript. "Related" means any relation with for-profit or not-for-profit third parties whose interests may be affected by the content of the manuscript. Disclosure represents a commitment to transparency and does not necessarily indicate a bias. If you are in doubt about whether to list a relationship/activity/interest, it is preferable that you do so.

The following questions apply to the author's relationships/activities/interests as they relate to the current manuscript only.

The author's relationships/activities/interests should be defined broadly. For example, if your manuscript pertains to the epidemiology of hypertension, you should declare all relationships with manufacturers of antihypertensive medication, even if that medication is not mentioned in the manuscript.

In item #1 below, report all support for the work reported in this manuscript without time limit. For all other items, the time frame for disclosure is the past 36 months.

		**Name all entities with whom you have this relationship or indicate none (add rows as needed)**	**Specifications/Comments (e.g., if payments were made to you or to your institution)**
		**Time frame: Since the initial planning of the work**	
1	All support for the present manuscript (e.g., funding, provision of study materials, medical writing, article processing charges, etc.) **No time limit for this item.**	_X_None	
		**Time frame: past 36 months**	
2	Grants or contracts from any entity (if not indicated in item #1 above).	_X__None	
3	Royalties or licenses	_X_None	
4	Consulting fees	_X_None	

5	Payment or honoraria for lectures, presentations, speakers bureaus, manuscript writing or educational events	__X_None	
6	Payment for expert testimony	_X__None	
7	Support for attending meetings and/or travel	_X_None	
8	Patents planned, issued or pending	_X_None	
9	Participation on a Data Safety Monitoring Board or Advisory Board	_X__None	
10	Leadership or fiduciary role in other board, society, committee or advocacy group, paid or unpaid	_X_None	
11	Stock or stock options	_X_None	
12	Receipt of equipment, materials, drugs, medical writing, gifts or other services	_X_None	
13	Other financial or non-financial interests	X__None	

Please place an "X" next to the following statement to indicate your agreement:

X_ I certify that I have answered every question and have not altered the wording of any of the questions on this form.

ICMJE DISCLOSURE FORM

Date:____03/03/2022____
Your Name:_____Eleanor Korendowych____
Manuscript Title:_ Comparative genetic analysis of psoriatic arthritis and psoriasis for the discovery of genetic risk factors and risk prediction modelling____
Manuscript number (if known):_ ar-21-1232____

In the interest of transparency, we ask you to disclose all relationships/activities/interests listed below that are related to the content of your manuscript. "Related" means any relation with for-profit or not-for-profit third parties whose interests may be affected by the content of the manuscript. Disclosure represents a commitment to transparency and does not necessarily indicate a bias. If you are in doubt about whether to list a relationship/activity/interest, it is preferable that you do so.

The following questions apply to the author's relationships/activities/interests as they relate to the current manuscript only.

The author's relationships/activities/interests should be defined broadly. For example, if your manuscript pertains to the epidemiology of hypertension, you should declare all relationships with manufacturers of antihypertensive medication, even if that medication is not mentioned in the manuscript.

In item #1 below, report all support for the work reported in this manuscript without time limit. For all other items, the time frame for disclosure is the past 36 months.

		**Name all entities with whom you have this relationship or indicate none (add rows as needed)**	**Specifications/Comments (e.g., if payments were made to you or to your institution)**
		**Time frame: Since the initial planning of the work**	
1	All support for the present manuscript (e.g., funding, provision of study materials, medical writing, article processing charges, etc.) **No time limit for this item.**	__X__None	
		**Time frame: past 36 months**	
2	Grants or contracts from any entity (if not indicated in item #1 above).	_X___None	
3	Royalties or licenses	__X__None	
4	Consulting fees	_X___None	

5	Payment or honoraria for lectures, presentations, speakers bureaus, manuscript writing or educational events	__X__None	
6	Payment for expert testimony	__X__None	
7	Support for attending meetings and/or travel	__X__None	
8	Patents planned, issued or pending	__X__None	
9	Participation on a Data Safety Monitoring Board or Advisory Board	__X__None	
10	Leadership or fiduciary role in other board, society, committee or advocacy group, paid or unpaid	__X__None	
11	Stock or stock options	__X__None	
12	Receipt of equipment, materials, drugs, medical writing, gifts or other services	__X__None	
13	Other financial or non-financial interests	__X__None	

Please place an "X" next to the following statement to indicate your agreement:

__X_ I certify that I have answered every question and have not altered the wording of any of the questions on this form.

ICMJE DISCLOSURE FORM

Date:______02/03/2022____
Your Name:______Farideh Jalali____
Manuscript Title:_ Comparative genetic analysis of psoriatic arthritis and psoriasis for the discovery of genetic risk factors and risk prediction modelling____
Manuscript number (if known):_ ar-21-1232____

In the interest of transparency, we ask you to disclose all relationships/activities/interests listed below that are related to the content of your manuscript. "Related" means any relation with for-profit or not-for-profit third parties whose interests may be affected by the content of the manuscript. Disclosure represents a commitment to transparency and does not necessarily indicate a bias. If you are in doubt about whether to list a relationship/activity/interest, it is preferable that you do so.

The following questions apply to the author's relationships/activities/interests as they relate to the <u>current manuscript only</u>.

The author's relationships/activities/interests should be <u>defined broadly</u>. For example, if your manuscript pertains to the epidemiology of hypertension, you should declare all relationships with manufacturers of antihypertensive medication, even if that medication is not mentioned in the manuscript.

In item #1 below, report all support for the work reported in this manuscript without time limit. For all other items, the time frame for disclosure is the past 36 months.

		**Name all entities with whom you have this relationship or indicate none (add rows as needed)**	**Specifications/Comments (e.g., if payments were made to you or to your institution)**
		**Time frame: Since the initial planning of the work**	
1	All support for the present manuscript (e.g., funding, provision of study materials, medical writing, article processing charges, etc.) **No time limit for this item.**	__X__None	
		**Time frame: past 36 months**	
2	Grants or contracts from any entity (if not indicated in item #1 above).	__X__None	
3	Royalties or licenses	__X__None	
4	Consulting fees	__X__None	

5	Payment or honoraria for lectures, presentations, speakers bureaus, manuscript writing or educational events	__X__None	
6	Payment for expert testimony	__X__None	
7	Support for attending meetings and/or travel	__X__None	
8	Patents planned, issued or pending	__X__None	
9	Participation on a Data Safety Monitoring Board or Advisory Board	__X__None	
10	Leadership or fiduciary role in other board, society, committee or advocacy group, paid or unpaid	__X__None	
11	Stock or stock options	__X__None	
12	Receipt of equipment, materials, drugs, medical writing, gifts or other services	_X___None	
13	Other financial or non-financial interests	__X__None	

Please place an "X" next to the following statement to indicate your agreement:

__X_ I certify that I have answered every question and have not altered the wording of any of the questions on this form.

ICMJE DISCLOSURE FORM

Date:______03/03/2022____
Your Name:_____Helena Marzo-Ortega____
Manuscript Title:_ Comparative genetic analysis of psoriatic arthritis and psoriasis for the discovery of genetic risk factors and risk prediction modelling____
Manuscript number (if known):_ ar-21-1232____

In the interest of transparency, we ask you to disclose all relationships/activities/interests listed below that are related to the content of your manuscript. "Related" means any relation with for-profit or not-for-profit third parties whose interests may be affected by the content of the manuscript. Disclosure represents a commitment to transparency and does not necessarily indicate a bias. If you are in doubt about whether to list a relationship/activity/interest, it is preferable that you do so.

The following questions apply to the author's relationships/activities/interests as they relate to the <u>current manuscript only</u>.

The author's relationships/activities/interests should be <u>defined broadly</u>. For example, if your manuscript pertains to the epidemiology of hypertension, you should declare all relationships with manufacturers of antihypertensive medication, even if that medication is not mentioned in the manuscript.

In item #1 below, report all support for the work reported in this manuscript without time limit. For all other items, the time frame for disclosure is the past 36 months.

		**Name all entities with whom you have this relationship or indicate none (add rows as needed)**	**Specifications/Comments (e.g., if payments were made to you or to your institution)**
**Time frame: Since the initial planning of the work**			
1	All support for the present manuscript (e.g., funding, provision of study materials, medical writing, article processing charges, etc.) **No time limit for this item.**	__X__None	
**Time frame: past 36 months**			
2	Grants or contracts from any entity (if not indicated in item #1 above).	__X__None	
3	Royalties or licenses	__X__None	
4	Consulting fees	__X__None	

5	Payment or honoraria for lectures, presentations, speakers bureaus, manuscript writing or educational events	__X__None	
6	Payment for expert testimony	__X__None	
7	Support for attending meetings and/or travel	__X__None	
8	Patents planned, issued or pending	__X__None	
9	Participation on a Data Safety Monitoring Board or Advisory Board	__X__None	
10	Leadership or fiduciary role in other board, society, committee or advocacy group, paid or unpaid	__X__None	
11	Stock or stock options	__X__None	
12	Receipt of equipment, materials, drugs, medical writing, gifts or other services	__X__None	
13	Other financial or non-financial interests	__X__None	

Please place an "X" next to the following statement to indicate your agreement:

__X_ I certify that I have answered every question and have not altered the wording of any of the questions on this form.

ICMJE DISCLOSURE FORM

Date:_______02/03/2022____
Your Name:___Jonathan Barker____
Manuscript Title:_ Comparative genetic analysis of psoriatic arthritis and psoriasis for the discovery of genetic risk factors and risk prediction modelling____
Manuscript number (if known):_ ar-21-1232____

In the interest of transparency, we ask you to disclose all relationships/activities/interests listed below that are related to the content of your manuscript. "Related" means any relation with for-profit or not-for-profit third parties whose interests may be affected by the content of the manuscript. Disclosure represents a commitment to transparency and does not necessarily indicate a bias. If you are in doubt about whether to list a relationship/activity/interest, it is preferable that you do so.

The following questions apply to the author's relationships/activities/interests as they relate to the current manuscript only.

The author's relationships/activities/interests should be defined broadly. For example, if your manuscript pertains to the epidemiology of hypertension, you should declare all relationships with manufacturers of antihypertensive medication, even if that medication is not mentioned in the manuscript.

In item #1 below, report all support for the work reported in this manuscript without time limit. For all other items, the time frame for disclosure is the past 36 months.

		**Name all entities with whom you have this relationship or indicate none (add rows as needed)**	**Specifications/Comments (e.g., if payments were made to you or to your institution)**
		**Time frame: Since the initial planning of the work**	
1	All support for the present manuscript (e.g., funding, provision of study materials, medical writing, article processing charges, etc.) **No time limit for this item.**	__X__None	
		**Time frame: past 36 months**	
2	Grants or contracts from any entity (if not indicated in item #1 above).	__X__None	
3	Royalties or licenses	__X__None	
4	Consulting fees	__X__None	

5	Payment or honoraria for lectures, presentations, speakers bureaus, manuscript writing or educational events	__X__None	
6	Payment for expert testimony	__X__None	
7	Support for attending meetings and/or travel	__X__None	
8	Patents planned, issued or pending	__X__None	
9	Participation on a Data Safety Monitoring Board or Advisory Board	__X__None	
10	Leadership or fiduciary role in other board, society, committee or advocacy group, paid or unpaid	_X___None	
11	Stock or stock options	__X__None	
12	Receipt of equipment, materials, drugs, medical writing, gifts or other services	__X__None	
13	Other financial or non-financial interests	__X__None	

Please place an "X" next to the following statement to indicate your agreement:

__X_ I certify that I have answered every question and have not altered the wording of any of the questions on this form.

31/01/2022 **ICMJE DISCLOSURE FORM**

Date:____
Your Name:___James Bluett____
Manuscript Title:_ Comparative genetic analysis of psoriatic arthritis and psoriasis for the discovery of genetic risk factors and risk prediction modelling____
Manuscript number (if known):_ ar-21-1232____

In the interest of transparency, we ask you to disclose all relationships/activities/interests listed below that are related to the content of your manuscript. "Related" means any relation with for-profit or not-for-profit third parties whose interests may be affected by the content of the manuscript. Disclosure represents a commitment to transparency and does not necessarily indicate a bias. If you are in doubt about whether to list a relationship/activity/interest, it is preferable that you do so.

The following questions apply to the author's relationships/activities/interests as they relate to the current manuscript only.

The author's relationships/activities/interests should be defined broadly. For example, if your manuscript pertains to the epidemiology of hypertension, you should declare all relationships with manufacturers of antihypertensive medication, even if that medication is not mentioned in the manuscript.

In item #1 below, report all support for the work reported in this manuscript without time limit. For all other items, the time frame for disclosure is the past 36 months.

		**Name all entities with whom you have this relationship or indicate none (add rows as needed)**	**Specifications/Comments (e.g., if payments were made to you or to your institution)**
		**Time frame: Since the initial planning of the work**	
1	All support for the present manuscript (e.g., funding, provision of study materials, medical writing, article processing charges, etc.) **No time limit for this item.**	Versus Arthritis	Grant funding to Institution
		NIHR	Grant funding to Institution
		**Time frame: past 36 months**	
2	Grants or contracts from any entity (if not indicated in item #1 above).	__X__None	
3	Royalties or licenses	__X__None	
4	Consulting fees	__X__None	

5	Payment or honoraria for lectures, presentations, speakers bureaus, manuscript writing or educational events	__X__None	
6	Payment for expert testimony	__X__None	
7	Support for attending meetings and/or travel	__X__None	
8	Patents planned, issued or pending	__X__None	
9	Participation on a Data Safety Monitoring Board or Advisory Board	__X__None	
10	Leadership or fiduciary role in other board, society, committee or advocacy group, paid or unpaid	__X__None	
11	Stock or stock options	__X__None	
12	Receipt of equipment, materials, drugs, medical writing, gifts or other services	__X__None	
13	Other financial or non-financial interests	__X__None	

Please place an "X" next to the following statement to indicate your agreement:

X_ I certify that I have answered every question and have not altered the wording of any of the questions on this form.

ICMJE DISCLOSURE FORM

Date:___09/02/2022____
Your Name:____John Bowes____
Manuscript Title:_ Comparative genetic analysis of psoriatic arthritis and psoriasis for the discovery of genetic risk factors and risk prediction modelling______________________________________
Manuscript number (if known):_ ar-21-1232____

In the interest of transparency, we ask you to disclose all relationships/activities/interests listed below that are related to the content of your manuscript. "Related" means any relation with for-profit or not-for-profit third parties whose interests may be affected by the content of the manuscript. Disclosure represents a commitment to transparency and does not necessarily indicate a bias. If you are in doubt about whether to list a relationship/activity/interest, it is preferable that you do so.

The following questions apply to the author's relationships/activities/interests as they relate to the current manuscript only.

The author's relationships/activities/interests should be defined broadly. For example, if your manuscript pertains to the epidemiology of hypertension, you should declare all relationships with manufacturers of antihypertensive medication, even if that medication is not mentioned in the manuscript.

In item #1 below, report all support for the work reported in this manuscript without time limit. For all other items, the time frame for disclosure is the past 36 months.

		**Name all entities with whom you have this relationship or indicate none (add rows as needed)**	**Specifications/Comments (e.g., if payments were made to you or to your institution)**
		**Time frame: Since the initial planning of the work**	
1	All support for the present manuscript (e.g., funding, provision of study materials, medical writing, article processing charges, etc.) **No time limit for this item.**	__X_None	
		**Time frame: past 36 months**	
2	Grants or contracts from any entity (if not indicated in item #1 above).	__X__None	
3	Royalties or licenses	__X__None	
4	Consulting fees	__X__None	

5	Payment or honoraria for lectures, presentations, speakers bureaus, manuscript writing or educational events	__X__None	
6	Payment for expert testimony	__X__None	
7	Support for attending meetings and/or travel	_X___None	
8	Patents planned, issued or pending	__X__None	
9	Participation on a Data Safety Monitoring Board or Advisory Board	__X__None	
10	Leadership or fiduciary role in other board, society, committee or advocacy group, paid or unpaid	__X__None	
11	Stock or stock options	__X__None	
12	Receipt of equipment, materials, drugs, medical writing, gifts or other services	__X__None	
13	Other financial or non-financial interests	__X__None	

Please place an "X" next to the following statement to indicate your agreement:

__X_ I certify that I have answered every question and have not altered the wording of any of the questions on this form.

ICMJE DISCLOSURE FORM

Date:________03/03/2022____
Your Name:_______Jonathan Packham____
Manuscript Title:_ Comparative genetic analysis of psoriatic arthritis and psoriasis for the discovery of genetic risk factors and risk prediction modelling____
Manuscript number (if known):_ ar-21-1232____

In the interest of transparency, we ask you to disclose all relationships/activities/interests listed below that are related to the content of your manuscript. "Related" means any relation with for-profit or not-for-profit third parties whose interests may be affected by the content of the manuscript. Disclosure represents a commitment to transparency and does not necessarily indicate a bias. If you are in doubt about whether to list a relationship/activity/interest, it is preferable that you do so.

The following questions apply to the author's relationships/activities/interests as they relate to the <u>current manuscript only</u>.

The author's relationships/activities/interests should be <u>defined broadly</u>. For example, if your manuscript pertains to the epidemiology of hypertension, you should declare all relationships with manufacturers of antihypertensive medication, even if that medication is not mentioned in the manuscript.

In item #1 below, report all support for the work reported in this manuscript without time limit. For all other items, the time frame for disclosure is the past 36 months.

		**Name all entities with whom you have this relationship or indicate none (add rows as needed)**	**Specifications/Comments (e.g., if payments were made to you or to your institution)**
		**Time frame: Since the initial planning of the work**	
1	All support for the present manuscript (e.g., funding, provision of study materials, medical writing, article processing charges, etc.) **No time limit for this item.**	_X_None	
		**Time frame: past 36 months**	
2	Grants or contracts from any entity (if not indicated in item #1 above).	__X__None	
3	Royalties or licenses	__X__None	
4	Consulting fees	__X__None	

5	Payment or honoraria for lectures, presentations, speakers bureaus, manuscript writing or educational events	__X__None	
6	Payment for expert testimony	__X__None	
7	Support for attending meetings and/or travel	__X__None	
8	Patents planned, issued or pending	__X__None	
9	Participation on a Data Safety Monitoring Board or Advisory Board	__X__None	
10	Leadership or fiduciary role in other board, society, committee or advocacy group, paid or unpaid	__X__None	
11	Stock or stock options	__X__None	
12	Receipt of equipment, materials, drugs, medical writing, gifts or other services	__X__None	
13	Other financial or non-financial interests	__X__None	

Please place an "X" next to the following statement to indicate your agreement:

_X__ I certify that I have answered every question and have not altered the wording of any of the questions on this form.

ICMJE DISCLOSURE FORM

Date:_______25/02/2022____
Your Name:________Matthew Brown____
Manuscript Title:_ Comparative genetic analysis of psoriatic arthritis and psoriasis for the discovery of genetic risk factors and risk prediction modelling____
Manuscript number (if known):_ ar-21-1232____

In the interest of transparency, we ask you to disclose all relationships/activities/interests listed below that are related to the content of your manuscript. "Related" means any relation with for-profit or not-for-profit third parties whose interests may be affected by the content of the manuscript. Disclosure represents a commitment to transparency and does not necessarily indicate a bias. If you are in doubt about whether to list a relationship/activity/interest, it is preferable that you do so.

The following questions apply to the author's relationships/activities/interests as they relate to the current manuscript only.

The author's relationships/activities/interests should be defined broadly. For example, if your manuscript pertains to the epidemiology of hypertension, you should declare all relationships with manufacturers of antihypertensive medication, even if that medication is not mentioned in the manuscript.

In item #1 below, report all support for the work reported in this manuscript without time limit. For all other items, the time frame for disclosure is the past 36 months.

		**Name all entities with whom you have this relationship or indicate none (add rows as needed)**	**Specifications/Comments (e.g., if payments were made to you or to your institution)**
		**Time frame: Since the initial planning of the work**	
1	All support for the present manuscript (e.g., funding, provision of study materials, medical writing, article processing charges, etc.) **No time limit for this item.**	Abbvie	Genotyping of PsA patients
		The National Health and Medical Research Council (Australia)	Senior Principal Research Fellowship
		**Time frame: past 36 months**	
2	Grants or contracts from any entity (if not indicated in item #1 above).	__X__None	
3	Royalties or licenses	__X__None	

4	Consulting fees	__X__None	
5	Payment or honoraria for lectures, presentations, speakers bureaus, manuscript writing or educational events	__X__None	
6	Payment for expert testimony	__X__None	
7	Support for attending meetings and/or travel	__X__None	
8	Patents planned, issued or pending	__X__None	
9	Participation on a Data Safety Monitoring Board or Advisory Board	_X___None	
10	Leadership or fiduciary role in other board, society, committee or advocacy group, paid or unpaid	__X__None	
11	Stock or stock options	__X__None	
12	Receipt of equipment, materials, drugs, medical writing, gifts or other services	__X__None	
13	Other financial or non-financial interests	__X__None	

Please place an "X" next to the following statement to indicate your agreement:

__X_ I certify that I have answered every question and have not altered the wording of any of the questions on this form.

ICMJE DISCLOSURE FORM

Date:______03/03/2022____
Your Name:______Michael Duckworth____
Manuscript Title:_ Comparative genetic analysis of psoriatic arthritis and psoriasis for the discovery of genetic risk factors and risk prediction modelling____
Manuscript number (if known):_ ar-21-1232____

In the interest of transparency, we ask you to disclose all relationships/activities/interests listed below that are related to the content of your manuscript. "Related" means any relation with for-profit or not-for-profit third parties whose interests may be affected by the content of the manuscript. Disclosure represents a commitment to transparency and does not necessarily indicate a bias. If you are in doubt about whether to list a relationship/activity/interest, it is preferable that you do so.

The following questions apply to the author's relationships/activities/interests as they relate to the current manuscript only.

The author's relationships/activities/interests should be defined broadly. For example, if your manuscript pertains to the epidemiology of hypertension, you should declare all relationships with manufacturers of antihypertensive medication, even if that medication is not mentioned in the manuscript.

In item #1 below, report all support for the work reported in this manuscript without time limit. For all other items, the time frame for disclosure is the past 36 months.

		**Name all entities with whom you have this relationship or indicate none (add rows as needed)**	**Specifications/Comments (e.g., if payments were made to you or to your institution)**
		**Time frame: Since the initial planning of the work**	
1	All support for the present manuscript (e.g., funding, provision of study materials, medical writing, article processing charges, etc.) **No time limit for this item.**	__X__None	
		**Time frame: past 36 months**	
2	Grants or contracts from any entity (if not indicated in item #1 above).	__X__None	
3	Royalties or licenses	__X__None	
4	Consulting fees	__X__None	

5	Payment or honoraria for lectures, presentations, speakers bureaus, manuscript writing or educational events	__X__None	
6	Payment for expert testimony	__X__None	
7	Support for attending meetings and/or travel	__X__None	
8	Patents planned, issued or pending	__X__None	
9	Participation on a Data Safety Monitoring Board or Advisory Board	__X__None	
10	Leadership or fiduciary role in other board, society, committee or advocacy group, paid or unpaid	__X__None	
11	Stock or stock options	__X__None	
12	Receipt of equipment, materials, drugs, medical writing, gifts or other services	__X__None	
13	Other financial or non-financial interests	__X__None	

Please place an "X" next to the following statement to indicate your agreement:

_x__ I certify that I have answered every question and have not altered the wording of any of the questions on this form.

ICMJE DISCLOSURE FORM

Date:____15.02.2022____
Your Name:_____Michael J. Nissen____
Manuscript Title:_ Comparative genetic analysis of psoriatic arthritis and psoriasis for the discovery of genetic risk factors and risk prediction modelling____
Manuscript number (if known):_ ar-21-1232____

In the interest of transparency, we ask you to disclose all relationships/activities/interests listed below that are related to the content of your manuscript. "Related" means any relation with for-profit or not-for-profit third parties whose interests may be affected by the content of the manuscript. Disclosure represents a commitment to transparency and does not necessarily indicate a bias. If you are in doubt about whether to list a relationship/activity/interest, it is preferable that you do so.

The following questions apply to the author's relationships/activities/interests as they relate to the current manuscript only.

The author's relationships/activities/interests should be defined broadly. For example, if your manuscript pertains to the epidemiology of hypertension, you should declare all relationships with manufacturers of antihypertensive medication, even if that medication is not mentioned in the manuscript.

In item #1 below, report all support for the work reported in this manuscript without time limit. For all other items, the time frame for disclosure is the past 36 months.

		**Name all entities with whom you have this relationship or indicate none (add rows as needed)**	**Specifications/Comments (e.g., if payments were made to you or to your institution)**
		**Time frame: Since the initial planning of the work**	
1	All support for the present manuscript (e.g., funding, provision of study materials, medical writing, article processing charges, etc.) **No time limit for this item.**	__X__None	
		**Time frame: past 36 months**	
2	Grants or contracts from any entity (if not indicated in item #1 above).	__X__None	
3	Royalties or licenses	__X__None	
4	Consulting fees		
		Celgene	Payment to my institution

5	Payment or honoraria for lectures, presentations, speakers bureaus, manuscript writing or educational events		
		Novartis	Presentation at an MRI workshop and symposium at a Swiss conference
		Eli Lilly	Presentation
6	Payment for expert testimony	__X__None	
7	Support for attending meetings and/or travel		
		UCB	Financial support to attend the Ghent SpA conference
8	Patents planned, issued or pending	__X__None	
9	Participation on a Data Safety Monitoring Board or Advisory Board		
		Janssen	Advisory Board
		Abbvie	Advisory Board
		Eli Lilly	Advisory Board
		Pfizer	Advisory Board
		Novartis	Advisory Board
10	Leadership or fiduciary role in other board, society, committee or advocacy group, paid or unpaid		
		SCQM committee member	Unpaid
		EuroSpA review board member	Unpaid
		EULAR task force member	Unpaid
11	Stock or stock options	__X__None	
12	Receipt of equipment, materials, drugs, medical writing, gifts or other services	__X__None	
13	Other financial or non-financial interests	__X__None	

Please place an "X" next to the following statement to indicate your agreement:

__X_ I certify that I have answered every question and have not altered the wording of any of the questions on this form.

ICMJE DISCLOSURE FORM

Date:_____03/03/2022____
Your Name:_____Michael Simpson____
Manuscript Title:_ Comparative genetic analysis of psoriatic arthritis and psoriasis for the discovery of genetic risk factors and risk prediction modelling____
Manuscript number (if known):_ ar-21-1232____

In the interest of transparency, we ask you to disclose all relationships/activities/interests listed below that are related to the content of your manuscript. "Related" means any relation with for-profit or not-for-profit third parties whose interests may be affected by the content of the manuscript. Disclosure represents a commitment to transparency and does not necessarily indicate a bias. If you are in doubt about whether to list a relationship/activity/interest, it is preferable that you do so.

The following questions apply to the author's relationships/activities/interests as they relate to the current manuscript only.

The author's relationships/activities/interests should be defined broadly. For example, if your manuscript pertains to the epidemiology of hypertension, you should declare all relationships with manufacturers of antihypertensive medication, even if that medication is not mentioned in the manuscript.

In item #1 below, report all support for the work reported in this manuscript without time limit. For all other items, the time frame for disclosure is the past 36 months.

		**Name all entities with whom you have this relationship or indicate none (add rows as needed)**	**Specifications/Comments (e.g., if payments were made to you or to your institution)**
		**Time frame: Since the initial planning of the work**	
1	All support for the present manuscript (e.g., funding, provision of study materials, medical writing, article processing charges, etc.) **No time limit for this item.**	__X__None	
		**Time frame: past 36 months**	
2	Grants or contracts from any entity (if not indicated in item #1 above).	__X__None	
3	Royalties or licenses	__X__None	
4	Consulting fees	__X__None	

5	Payment or honoraria for lectures, presentations, speakers bureaus, manuscript writing or educational events	__X__None	
6	Payment for expert testimony	__X__None	
7	Support for attending meetings and/or travel	__X__None	
8	Patents planned, issued or pending	__X__None	
9	Participation on a Data Safety Monitoring Board or Advisory Board	__X__None	
10	Leadership or fiduciary role in other board, society, committee or advocacy group, paid or unpaid	__X__None	
11	Stock or stock options	__X__None	
12	Receipt of equipment, materials, drugs, medical writing, gifts or other services	__X__None	
13	Other financial or non-financial interests	_X__None	

Please place an "X" next to the following statement to indicate your agreement:

__X_ I certify that I have answered every question and have not altered the wording of any of the questions on this form.

ICMJE DISCLOSURE FORM

Date:___1-02-22____
Your Name:_MEHREEN SOOMRO____
Manuscript Title:_ Comparative genetic analysis of psoriatic arthritis and psoriasis for the discovery of genetic risk factors and risk prediction modelling____
Manuscript number (if known):_ ar-21-1232____

In the interest of transparency, we ask you to disclose all relationships/activities/interests listed below that are related to the content of your manuscript. "Related" means any relation with for-profit or not-for-profit third parties whose interests may be affected by the content of the manuscript. Disclosure represents a commitment to transparency and does not necessarily indicate a bias. If you are in doubt about whether to list a relationship/activity/interest, it is preferable that you do so.

The following questions apply to the author's relationships/activities/interests as they relate to the current manuscript only.

The author's relationships/activities/interests should be defined broadly. For example, if your manuscript pertains to the epidemiology of hypertension, you should declare all relationships with manufacturers of antihypertensive medication, even if that medication is not mentioned in the manuscript.

In item #1 below, report all support for the work reported in this manuscript without time limit. For all other items, the time frame for disclosure is the past 36 months.

		**Name all entities with whom you have this relationship or indicate none (add rows as needed)**	**Specifications/Comments (e.g., if payments were made to you or to your institution)**
		**Time frame: Since the initial planning of the work**	
1	All support for the present manuscript (e.g., funding, provision of study materials, medical writing, article processing charges, etc.) **No time limit for this item.**	__X__None	
		**Time frame: past 36 months**	
2	Grants or contracts from any entity (if not indicated in item #1 above).	__X__None	
3	Royalties or licenses	__X__None	
4	Consulting fees	__X__None	

5	Payment or honoraria for lectures, presentations, speakers bureaus, manuscript writing or educational events	__X__None	
6	Payment for expert testimony	__X__None	
7	Support for attending meetings and/or travel	__X__None	
8	Patents planned, issued or pending	__X__None	
9	Participation on a Data Safety Monitoring Board or Advisory Board	__X__None	
10	Leadership or fiduciary role in other board, society, committee or advocacy group, paid or unpaid	__X__None	
11	Stock or stock options	__X__None	
12	Receipt of equipment, materials, drugs, medical writing, gifts or other services	__X__None	
13	Other financial or non-financial interests	__X__None	

Please place an "X" next to the following statement to indicate your agreement:

__X_ I certify that I have answered every question and have not altered the wording of any of the questions on this form.

ICMJE DISCLOSURE FORM

Date:___09/02/2022____
Your Name:____Michael Stadler____
Manuscript Title:_ Comparative genetic analysis of psoriatic arthritis and psoriasis for the discovery of genetic risk factors and risk prediction modelling____
Manuscript number (if known):_ ar-21-1232____

In the interest of transparency, we ask you to disclose all relationships/activities/interests listed below that are related to the content of your manuscript. "Related" means any relation with for-profit or not-for-profit third parties whose interests may be affected by the content of the manuscript. Disclosure represents a commitment to transparency and does not necessarily indicate a bias. If you are in doubt about whether to list a relationship/activity/interest, it is preferable that you do so.

The following questions apply to the author's relationships/activities/interests as they relate to the current manuscript only.

The author's relationships/activities/interests should be defined broadly. For example, if your manuscript pertains to the epidemiology of hypertension, you should declare all relationships with manufacturers of antihypertensive medication, even if that medication is not mentioned in the manuscript.

In item #1 below, report all support for the work reported in this manuscript without time limit. For all other items, the time frame for disclosure is the past 36 months.

		**Name all entities with whom you have this relationship or indicate none (add rows as needed)**	**Specifications/Comments (e.g., if payments were made to you or to your institution)**
		**Time frame: Since the initial planning of the work**	
1	All support for the present manuscript (e.g., funding, provision of study materials, medical writing, article processing charges, etc.) **No time limit for this item.**	__X__None	
		**Time frame: past 36 months**	
2	Grants or contracts from any entity (if not indicated in item #1 above).	__X__None	
3	Royalties or licenses	__X__None	
4	Consulting fees	__X__None	

5	Payment or honoraria for lectures, presentations, speakers bureaus, manuscript writing or educational events	__X__None	
6	Payment for expert testimony	__X__None	
7	Support for attending meetings and/or travel	__X__None	
8	Patents planned, issued or pending	__X__None	
9	Participation on a Data Safety Monitoring Board or Advisory Board	__X__None	
10	Leadership or fiduciary role in other board, society, committee or advocacy group, paid or unpaid	__X__None	
11	Stock or stock options	__X__None	
12	Receipt of equipment, materials, drugs, medical writing, gifts or other services	__X__None	
13	Other financial or non-financial interests	__X__None	

Please place an "X" next to the following statement to indicate your agreement:

__X_ I certify that I have answered every question and have not altered the wording of any of the questions on this form.

ICMJE DISCLOSURE FORM

Date:_____03/03/2022____
Your Name:____Nick Dand____
Manuscript Title:_ Comparative genetic analysis of psoriatic arthritis and psoriasis for the discovery of genetic risk factors and risk prediction modelling____
Manuscript number (if known):_ ar-21-1232____

In the interest of transparency, we ask you to disclose all relationships/activities/interests listed below that are related to the content of your manuscript. "Related" means any relation with for-profit or not-for-profit third parties whose interests may be affected by the content of the manuscript. Disclosure represents a commitment to transparency and does not necessarily indicate a bias. If you are in doubt about whether to list a relationship/activity/interest, it is preferable that you do so.

The following questions apply to the author's relationships/activities/interests as they relate to the current manuscript only.

The author's relationships/activities/interests should be defined broadly. For example, if your manuscript pertains to the epidemiology of hypertension, you should declare all relationships with manufacturers of antihypertensive medication, even if that medication is not mentioned in the manuscript.

In item #1 below, report all support for the work reported in this manuscript without time limit. For all other items, the time frame for disclosure is the past 36 months.

		**Name all entities with whom you have this relationship or indicate none (add rows as needed)**	**Specifications/Comments (e.g., if payments were made to you or to your institution)**
		**Time frame: Since the initial planning of the work**	
1	All support for the present manuscript (e.g., funding, provision of study materials, medical writing, article processing charges, etc.) **No time limit for this item.**	__X__None	
		**Time frame: past 36 months**	
2	Grants or contracts from any entity (if not indicated in item #1 above).	__X__None	
3	Royalties or licenses	__X__None	
4	Consulting fees	__X__None	

5	Payment or honoraria for lectures, presentations, speakers bureaus, manuscript writing or educational events	__X__None	
6	Payment for expert testimony	__X__None	
7	Support for attending meetings and/or travel	__X__None	
8	Patents planned, issued or pending	__X__None	
9	Participation on a Data Safety Monitoring Board or Advisory Board	__X__None	
10	Leadership or fiduciary role in other board, society, committee or advocacy group, paid or unpaid	__X__None	
11	Stock or stock options	__X__None	
12	Receipt of equipment, materials, drugs, medical writing, gifts or other services	__X__None	
13	Other financial or non-financial interests	__X__None	

Please place an "X" next to the following statement to indicate your agreement:

_x__ I certify that I have answered every question and have not altered the wording of any of the questions on this form.

ICMJE DISCLOSURE FORM

Date:________03/03/2022____
Your Name:____Neil McHugh____
Manuscript Title:_ Comparative genetic analysis of psoriatic arthritis and psoriasis for the discovery of genetic risk factors and risk prediction modelling____
Manuscript number (if known):_ ar-21-1232____

In the interest of transparency, we ask you to disclose all relationships/activities/interests listed below that are related to the content of your manuscript. "Related" means any relation with for-profit or not-for-profit third parties whose interests may be affected by the content of the manuscript. Disclosure represents a commitment to transparency and does not necessarily indicate a bias. If you are in doubt about whether to list a relationship/activity/interest, it is preferable that you do so.

The following questions apply to the author's relationships/activities/interests as they relate to the current manuscript only.

The author's relationships/activities/interests should be defined broadly. For example, if your manuscript pertains to the epidemiology of hypertension, you should declare all relationships with manufacturers of antihypertensive medication, even if that medication is not mentioned in the manuscript.

In item #1 below, report all support for the work reported in this manuscript without time limit. For all other items, the time frame for disclosure is the past 36 months.

		**Name all entities with whom you have this relationship or indicate none (add rows as needed)**	**Specifications/Comments (e.g., if payments were made to you or to your institution)**
		**Time frame: Since the initial planning of the work**	
1	All support for the present manuscript (e.g., funding, provision of study materials, medical writing, article processing charges, etc.) **No time limit for this item.**	__X__None	
		**Time frame: past 36 months**	
2	Grants or contracts from any entity (if not indicated in item #1 above).	__X__None	
3	Royalties or licenses	__X__None	
4	Consulting fees	__X__None	

5	Payment or honoraria for lectures, presentations, speakers bureaus, manuscript writing or educational events	__X__None	
6	Payment for expert testimony	__X__None	
7	Support for attending meetings and/or travel	__X__None	
8	Patents planned, issued or pending	__X__None	
9	Participation on a Data Safety Monitoring Board or Advisory Board	__X__None	
10	Leadership or fiduciary role in other board, society, committee or advocacy group, paid or unpaid	__X__None	
11	Stock or stock options	__X__None	
12	Receipt of equipment, materials, drugs, medical writing, gifts or other services	__X__None	
13	Other financial or non-financial interests	__X__None	

Please place an "X" next to the following statement to indicate your agreement:

__X_ I certify that I have answered every question and have not altered the wording of any of the questions on this form.

ICMJE DISCLOSURE FORM

Date:______03/03/2022____
Your Name:___Oliver FitzGerald____
Manuscript Title:_ Comparative genetic analysis of psoriatic arthritis and psoriasis for the discovery of genetic risk factors and risk prediction modelling____
Manuscript number (if known):_ ar-21-1232____

In the interest of transparency, we ask you to disclose all relationships/activities/interests listed below that are related to the content of your manuscript. "Related" means any relation with for-profit or not-for-profit third parties whose interests may be affected by the content of the manuscript. Disclosure represents a commitment to transparency and does not necessarily indicate a bias. If you are in doubt about whether to list a relationship/activity/interest, it is preferable that you do so.

The following questions apply to the author's relationships/activities/interests as they relate to the current manuscript only.

The author's relationships/activities/interests should be defined broadly. For example, if your manuscript pertains to the epidemiology of hypertension, you should declare all relationships with manufacturers of antihypertensive medication, even if that medication is not mentioned in the manuscript.

In item #1 below, report all support for the work reported in this manuscript without time limit. For all other items, the time frame for disclosure is the past 36 months.

		**Name all entities with whom you have this relationship or indicate none (add rows as needed)**	**Specifications/Comments (e.g., if payments were made to you or to your institution)**
		**Time frame: Since the initial planning of the work**	
1	All support for the present manuscript (e.g., funding, provision of study materials, medical writing, article processing charges, etc.) **No time limit for this item.**	__X__None	
		**Time frame: past 36 months**	
2	Grants or contracts from any entity (if not indicated in item #1 above).	__X__None	
3	Royalties or licenses	__X__None	
4	Consulting fees	__X__None	

5	Payment or honoraria for lectures, presentations, speakers bureaus, manuscript writing or educational events	__X__None	
6	Payment for expert testimony	__X__None	
7	Support for attending meetings and/or travel	__X__None	
8	Patents planned, issued or pending	__X__None	
9	Participation on a Data Safety Monitoring Board or Advisory Board	__X__None	
10	Leadership or fiduciary role in other board, society, committee or advocacy group, paid or unpaid	__X__None	
11	Stock or stock options	__X__None	
12	Receipt of equipment, materials, drugs, medical writing, gifts or other services	__X__None	
13	Other financial or non-financial interests	__X__None	

Please place an "X" next to the following statement to indicate your agreement:

__X_ I certify that I have answered every question and have not altered the wording of any of the questions on this form.

ICMJE DISCLOSURE FORM

Date:____10th February 2022____
Your Name:___Philip S Helliwell____
Manuscript Title:_ Comparative genetic analysis of psoriatic arthritis and psoriasis for the discovery of genetic risk factors and risk prediction modelling____
Manuscript number (if known):_ ar-21-1232____

In the interest of transparency, we ask you to disclose all relationships/activities/interests listed below that are related to the content of your manuscript. "Related" means any relation with for-profit or not-for-profit third parties whose interests may be affected by the content of the manuscript. Disclosure represents a commitment to transparency and does not necessarily indicate a bias. If you are in doubt about whether to list a relationship/activity/interest, it is preferable that you do so.

The following questions apply to the author's relationships/activities/interests as they relate to the current manuscript only.

The author's relationships/activities/interests should be defined broadly. For example, if your manuscript pertains to the epidemiology of hypertension, you should declare all relationships with manufacturers of antihypertensive medication, even if that medication is not mentioned in the manuscript.

In item #1 below, report all support for the work reported in this manuscript without time limit. For all other items, the time frame for disclosure is the past 36 months.

		**Name all entities with whom you have this relationship or indicate none (add rows as needed)**	**Specifications/Comments (e.g., if payments were made to you or to your institution)**
		**Time frame: Since the initial planning of the work**	
1	All support for the present manuscript (e.g., funding, provision of study materials, medical writing, article processing charges, etc.) **No time limit for this item.**	__X__None	
		**Time frame: past 36 months**	
2	Grants or contracts from any entity (if not indicated in item #1 above).	__X__None	
3	Royalties or licenses	__X__None	
4	Consulting fees	____Pfizer and Lilly	

5	Payment or honoraria for lectures, presentations, speakers bureaus, manuscript writing or educational events	____Novartis, Abbvie and Janssen	
6	Payment for expert testimony	__X__None	
7	Support for attending meetings and/or travel	__X__None	
8	Patents planned, issued or pending	__X__None	
9	Participation on a Data Safety Monitoring Board or Advisory Board	__X__None	
10	Leadership or fiduciary role in other board, society, committee or advocacy group, paid or unpaid	__X__None	
11	Stock or stock options	__X__None	
12	Receipt of equipment, materials, drugs, medical writing, gifts or other services	__X__None	
13	Other financial or non-financial interests	__X__None	

Please place an "X" next to the following statement to indicate your agreement:

__x_ I certify that I have answered every question and have not altered the wording of any of the questions on this form.

ICMJE DISCLOSURE FORM

Date:________02/03/2022____
Your Name:______Pauline Ho____
Manuscript Title:_ Comparative genetic analysis of psoriatic arthritis and psoriasis for the discovery of genetic risk factors and risk prediction modelling____
Manuscript number (if known):_ ar-21-1232____

In the interest of transparency, we ask you to disclose all relationships/activities/interests listed below that are related to the content of your manuscript. "Related" means any relation with for-profit or not-for-profit third parties whose interests may be affected by the content of the manuscript. Disclosure represents a commitment to transparency and does not necessarily indicate a bias. If you are in doubt about whether to list a relationship/activity/interest, it is preferable that you do so.

The following questions apply to the author's relationships/activities/interests as they relate to the current manuscript only.

The author's relationships/activities/interests should be defined broadly. For example, if your manuscript pertains to the epidemiology of hypertension, you should declare all relationships with manufacturers of antihypertensive medication, even if that medication is not mentioned in the manuscript.

In item #1 below, report all support for the work reported in this manuscript without time limit. For all other items, the time frame for disclosure is the past 36 months.

		**Name all entities with whom you have this relationship or indicate none (add rows as needed)**	**Specifications/Comments (e.g., if payments were made to you or to your institution)**
		**Time frame: Since the initial planning of the work**	
1	All support for the present manuscript (e.g., funding, provision of study materials, medical writing, article processing charges, etc.) **No time limit for this item.**	__X__None	
		**Time frame: past 36 months**	
2	Grants or contracts from any entity (if not indicated in item #1 above).	__X__None	
3	Royalties or licenses	__X__None	
4	Consulting fees	__X__None	

5	Payment or honoraria for lectures, presentations, speakers bureaus, manuscript writing or educational events	__X__None	
6	Payment for expert testimony	__X__None	
7	Support for attending meetings and/or travel	__X__None	
8	Patents planned, issued or pending	__X__None	
9	Participation on a Data Safety Monitoring Board or Advisory Board	__X__None	
10	Leadership or fiduciary role in other board, society, committee or advocacy group, paid or unpaid	__X__None	
11	Stock or stock options	__X__None	
12	Receipt of equipment, materials, drugs, medical writing, gifts or other services	__X__None	
13	Other financial or non-financial interests	__X__None	

Please place an "X" next to the following statement to indicate your agreement:

___X I certify that I have answered every question and have not altered the wording of any of the questions on this form.

ICMJE DISCLOSURE FORM

Date:____25 February 2022 ____
Your Name:___ Ross McManus ____
Manuscript Title:_ Comparative genetic analysis of psoriatic arthritis and psoriasis for the discovery of genetic risk factors and risk prediction modelling____
Manuscript number (if known):_ ar-21-1232____

In the interest of transparency, we ask you to disclose all relationships/activities/interests listed below that are related to the content of your manuscript. "Related" means any relation with for-profit or not-for-profit third parties whose interests may be affected by the content of the manuscript. Disclosure represents a commitment to transparency and does not necessarily indicate a bias. If you are in doubt about whether to list a relationship/activity/interest, it is preferable that you do so.

The following questions apply to the author's relationships/activities/interests as they relate to the current manuscript only.

The author's relationships/activities/interests should be defined broadly. For example, if your manuscript pertains to the epidemiology of hypertension, you should declare all relationships with manufacturers of antihypertensive medication, even if that medication is not mentioned in the manuscript.

In item #1 below, report all support for the work reported in this manuscript without time limit. For all other items, the time frame for disclosure is the past 36 months.

		**Name all entities with whom you have this relationship or indicate none (add rows as needed)**	**Specifications/Comments (e.g., if payments were made to you or to your institution)**
		**Time frame: Since the initial planning of the work**	
1	All support for the present manuscript (e.g., funding, provision of study materials, medical writing, article processing charges, etc.) **No time limit for this item.**	__X__None	
		**Time frame: past 36 months**	
2	Grants or contracts from any entity (if not indicated in item #1 above).	__X__None	
3	Royalties or licenses	__X__None	
4	Consulting fees	__X__None	

5	Payment or honoraria for lectures, presentations, speakers bureaus, manuscript writing or educational events	__X__None	
6	Payment for expert testimony	__X__None	
7	Support for attending meetings and/or travel	__X__None	
8	Patents planned, issued or pending	__X__None	
9	Participation on a Data Safety Monitoring Board or Advisory Board	__X__None	
10	Leadership or fiduciary role in other board, society, committee or advocacy group, paid or unpaid	__X__None	
11	Stock or stock options	__X__None	
12	Receipt of equipment, materials, drugs, medical writing, gifts or other services	__X__None	
13	Other financial or non-financial interests	__X__None	

Please place an "X" next to the following statement to indicate your agreement:

__X_ I certify that I have answered every question and have not altered the wording of any of the questions on this form.

ICMJE DISCLOSURE FORM

Date: 01/02/2022
Your Name: Professor Richard Warren
Manuscript Title:_ Comparative genetic analysis of psoriatic arthritis and psoriasis for the discovery of genetic risk factors and risk prediction modelling____
Manuscript number (if known):_ ar-21-1232____

In the interest of transparency, we ask you to disclose all relationships/activities/interests listed below that are related to the content of your manuscript. "Related" means any relation with for-profit or not-for-profit third parties whose interests may be affected by the content of the manuscript. Disclosure represents a commitment to transparency and does not necessarily indicate a bias. If you are in doubt about whether to list a relationship/activity/interest, it is preferable that you do so.

The following questions apply to the author's relationships/activities/interests as they relate to the current manuscript only.

The author's relationships/activities/interests should be defined broadly. For example, if your manuscript pertains to the epidemiology of hypertension, you should declare all relationships with manufacturers of antihypertensive medication, even if that medication is not mentioned in the manuscript.

In item #1 below, report all support for the work reported in this manuscript without time limit. For all other items, the time frame for disclosure is the past 36 months.

		**Name all entities with whom you have this relationship or indicate none (add rows as needed)**	**Specifications/Comments (e.g., if payments were made to you or to your institution)**
**Time frame: Since the initial planning of the work**			
1	All support for the present manuscript (e.g., funding, provision of study materials, medical writing, article processing charges, etc.) **No time limit for this item.**	__X__None	
**Time frame: past 36 months**			
2	Grants or contracts from any entity (if not indicated in item #1 above).	AbbVie, Almirall, Janssen, Lilly, Leo, Novartis, Pfizer & UCB.	
3	Royalties or licenses	_X__None	
4	Consulting fees	AbbVie, Almirall, Amgen, Arena,	

		Astellas, Avillion, Biogen, Boehringer Ingelheim, Bristol Myers Squibb, Celgene, DiCE, GSK, Janssen, Lilly, Leo, Novartis, Pfizer, Sanofi, Sun Pharma, UCB & UNION.	
5	Payment or honoraria for lectures, presentations, speakers bureaus, manuscript writing or educational events	AbbVie, Almirall, , Boehringer Ingelheim, Bristol Myers Squibb, Celgene, Janssen, Lilly, Leo, Novartis, Pfizer, Sanofi, Sun Pharma & UCB.	
6	Payment for expert testimony	__X__None	
7	Support for attending meetings and/or travel	__X__None	
8	Patents planned, issued or pending	__X__None	
9	Participation on a Data Safety Monitoring Board or Advisory Board	__X__None	
10	Leadership or fiduciary role in other board, society, committee or advocacy group, paid or unpaid	__X__None	
11	Stock or stock options	__X__None	
12	Receipt of equipment, materials, drugs, medical writing, gifts or other services	__X__None	
13	Other financial or non-financial interests	__X__None	

Please place an "X" next to the following statement to indicate your agreement:

X I certify that I have answered every question and have not altered the wording of any of the questions on this form.

ICMJE DISCLOSURE FORM

Date:________02/03/2022____
Your Name:______Suzanne Verstappen____
Manuscript Title:_ Comparative genetic analysis of psoriatic arthritis and psoriasis for the discovery of genetic risk factors and risk prediction modelling____
Manuscript number (if known):_ ar-21-1232____

In the interest of transparency, we ask you to disclose all relationships/activities/interests listed below that are related to the content of your manuscript. "Related" means any relation with for-profit or not-for-profit third parties whose interests may be affected by the content of the manuscript. Disclosure represents a commitment to transparency and does not necessarily indicate a bias. If you are in doubt about whether to list a relationship/activity/interest, it is preferable that you do so.

The following questions apply to the author's relationships/activities/interests as they relate to the current manuscript only.

The author's relationships/activities/interests should be defined broadly. For example, if your manuscript pertains to the epidemiology of hypertension, you should declare all relationships with manufacturers of antihypertensive medication, even if that medication is not mentioned in the manuscript.

In item #1 below, report all support for the work reported in this manuscript without time limit. For all other items, the time frame for disclosure is the past 36 months.

		**Name all entities with whom you have this relationship or indicate none (add rows as needed)**	**Specifications/Comments (e.g., if payments were made to you or to your institution)**
		**Time frame: Since the initial planning of the work**	
1	All support for the present manuscript (e.g., funding, provision of study materials, medical writing, article processing charges, etc.) **No time limit for this item.**	__X__None	
		**Time frame: past 36 months**	
2	Grants or contracts from any entity (if not indicated in item #1 above).	__X__None	
3	Royalties or licenses	__X__None	
4	Consulting fees	__X__None	

5	Payment or honoraria for lectures, presentations, speakers bureaus, manuscript writing or educational events	__X__None	
6	Payment for expert testimony	__X__None	
7	Support for attending meetings and/or travel	__X__None	
8	Patents planned, issued or pending	__X__None	
9	Participation on a Data Safety Monitoring Board or Advisory Board	__X__None	
10	Leadership or fiduciary role in other board, society, committee or advocacy group, paid or unpaid	__X__None	
11	Stock or stock options	__X__None	
12	Receipt of equipment, materials, drugs, medical writing, gifts or other services	__X__None	
13	Other financial or non-financial interests	__X__None	

Please place an "X" next to the following statement to indicate your agreement:

___X I certify that I have answered every question and have not altered the wording of any of the questions on this form.

ICMJE DISCLOSURE FORM

Date:_25-2-2022____
Your Name:___Tjeerd van Staa____
Manuscript Title:_ Comparative genetic analysis of psoriatic arthritis and psoriasis for the discovery of genetic risk factors and risk prediction modelling____
Manuscript number (if known):_ ar-21-1232____

In the interest of transparency, we ask you to disclose all relationships/activities/interests listed below that are related to the content of your manuscript. "Related" means any relation with for-profit or not-for-profit third parties whose interests may be affected by the content of the manuscript. Disclosure represents a commitment to transparency and does not necessarily indicate a bias. If you are in doubt about whether to list a relationship/activity/interest, it is preferable that you do so.

The following questions apply to the author's relationships/activities/interests as they relate to the <u>current manuscript only</u>.

The author's relationships/activities/interests should be <u>defined broadly</u>. For example, if your manuscript pertains to the epidemiology of hypertension, you should declare all relationships with manufacturers of antihypertensive medication, even if that medication is not mentioned in the manuscript.

In item #1 below, report all support for the work reported in this manuscript without time limit. For all other items, the time frame for disclosure is the past 36 months.

		**Name all entities with whom you have this relationship or indicate none (add rows as needed)**	**Specifications/Comments (e.g., if payments were made to you or to your institution)**
**Time frame: Since the initial planning of the work**			
1	All support for the present manuscript (e.g., funding, provision of study materials, medical writing, article processing charges, etc.) **No time limit for this item.**	_X_None	
**Time frame: past 36 months**			
2	Grants or contracts from any entity (if not indicated in item #1 above).	_X_None	
3	Royalties or licenses	_X_None	
4	Consulting fees	_X_None	

5	Payment or honoraria for lectures, presentations, speakers bureaus, manuscript writing or educational events	_X_None	
6	Payment for expert testimony	_X_None	
7	Support for attending meetings and/or travel	_X_None	
8	Patents planned, issued or pending	_X_None	
9	Participation on a Data Safety Monitoring Board or Advisory Board	_X_None	
10	Leadership or fiduciary role in other board, society, committee or advocacy group, paid or unpaid	_X_None	
11	Stock or stock options	_X_None	
12	Receipt of equipment, materials, drugs, medical writing, gifts or other services	_X_None	
13	Other financial or non-financial interests	_X_None	

Please place an "X" next to the following statement to indicate your agreement:

_X__ I certify that I have answered every question and have not altered the wording of any of the questions on this form.
