# Supplementary material for: Comparative Genetic Analysis of Psoriatic Arthritis and Psoriasis for the Discovery of Genetic Risk Factors and Risk Prediction Modeling
Source: Arthritis Rheumatol. 2022 Aug 4;74(9):1535–43. doi: 10.1002/art.42154 (PMC9539852; doi:10.1002/art.42154)
Supplement: Supplementary file 2 — Appendix S1 Supporting Information [file ART-74-1535-s001.docx]

**Supplementary material**

Contents

[Supplementary methods 2](#_Toc95306185)

[Statistical quality control 2](#_Toc95306186)

[Association testing and meta-analysis 2](#_Toc95306187)

[Heritability estimates 3](#_Toc95306188)

[Risk prediction 3](#_Toc95306189)

[Supplementary figures 5](#_Toc95306190)

[Supplementary Figure 1: summary of clinical data from the BSTOP cohort 5](#_Toc95306191)

[Supplementary Figure 2: genotyping and quality control of the PsA-BSTOP dataset. 6](#_Toc95306192)

[Supplementary Figure 3: summary of reported meta-analyses 7](#_Toc95306193)

[Supplementary Figure 4: summary of prediction analyses 8](#_Toc95306194)

[Supplementary Figure 5: calibration curve for the random forest model 9](#_Toc95306195)

[Supplementary Figure 6: calibration curve for the conditional inference forest model 10](#_Toc95306196)

[Supplementary figure 7: SparSNP AUC results 11](#_Toc95306197)

[Supplementary figure 8: SparSNP calibration curve 11](#_Toc95306198)

# Supplementary methods

## Statistical quality control

Poor quality PsA DNA samples (call rate < 0.90) were excluded using the default Illumina cluster file followed by automated reclustering to calibrate genotype clusters on the study samples. Sample-level quality control (QC) excluded samples with a call rate < 0.98, outliers based on autosomal heterozygosity (2 standard deviations from the mean) and discrepancy between genetically inferred sex and database records. SNP QC excluded non-autosomal SNPs, those with a call rate < 0.98 or a minor allele frequency < 0.01.

The three datasets (PsA, PsC and controls) were combined retaining the intersection of SNPs and further QC was performed with Identity-by-descent (IBD) which was utilised to identify related individuals (kinship coefficient > 0.0884) across all study samples performed with the KING software package (version 1.9)[15]. The sample with the highest call rate was preferentially retained for each related pair. Individuals were excluded if they were identified as outliers, based on ancestry using principal component analysis (PCA) performed with the flashpca software package (version 2.0) where outliers with atypical ancestry, based on the top two principal components, were identified using the aberrant clustering algorithm R library (version 1.0)[16,17].

## Association testing and meta-analysis

Case-control association testing in the PsA-BSTOP and UK Biobank self-reported datasets was performed using the SNPTEST software package (version 2.5.2) using the score method to account for imputation uncertainty. Two association tests were conducted in each of these two datasets; PsA compared to population controls and PsA to PsC. Three principal components, calculated as described above, were included as covariates to account for any residual population structure. A meta-analysis of summary statistics from the PsA-BSTOP, UK Biobank and the PsA Immunochip datasets was performed for PsA compared to population controls. A separate meta-analysis was conducted of summary statistics from the PsA-BSTOP and UK Biobank datasets comparing PsA to PsC. These were conducted using an inverse variance meta-analysis assuming fixed effects with the software package GWAMA (version 2.2.2)[20]. Between study heterogeneity of odds ratios was measured with Cochran’s Q statistic and the I^2^ index. Lambda genomic control (λGC), corrected for sample size (λGC_1000_), was calculated to test for inflation of test statistics attributable to population stratification, and potential test statistic inflation from other sources.

## Heritability estimates

Heritability of PsA and PsC was estimated in the PsA-BSTOP GWAS dataset using GCTA. SNPs were stratified into quartiles based on linkage disequilibrium (LD) then further stratified into minor allele frequency (MAF) bins (0.01 < MAF ≤ 0.1; 0.1 < MAF ≤ 0.2, 0.2 < MAF ≤ 0.3; 0.3 < MAF ≤ 0.4; 0.4 < MAF ≤ 0.5) to create 20 genetic relationship matrices[21]. Phenotypic variance attributed to imputed SNPs was then estimated using GREML with three principal components as covariates[22]. Estimates were calculated with no prevalence specified and repeated with a specified disease prevalence of 1% for comparison with previously reported estimates[7]. Both estimates were re-calculated excluding SNPs from the major histocompatibility complex (MHC).

## Risk prediction

The overall model performance was further assessed in the UK Biobank ICD10 dataset as an external validation dataset using the Brier score and c-statistic. The Brier score is the squared difference between predicted and observed risk and ranges from 0 for a perfect model to 0.25 for an uninformative model which has 50 percent incidence of the outcome. Under an uninformative model, it can be scaled by maximum Brier score, so that it ranges from 0 to 100%. We further assessed the model calibration using simple logistic regression to calculate the difference in the log-odds between predictions and outcomes, also called ‘calibration in the large’ (CITL (intercept(a)), to check for systematically low or high predictions; the model’s general fit was then determined using the calibration slope (b). Calibration (seen on a scatter plot) represents the relationship between observed (y-axis) and predicted risk (x-axis), and perfect predictions should lie on the 45° line. In model development, a = 0 and b =1, however, in independent validation, CITL problems are defined if a ≠ 0, and if b is greater/less than 1, as this shows under/overprediction issues respectively. Lastly, we evaluated the clinical utility of the models using common performance metrics derived from a confusion matrix of correct and false predictions per class to calculate the model’s overall accuracy, precision, sensitivity, specificity, and the negative predictive value.

# Supplementary figures


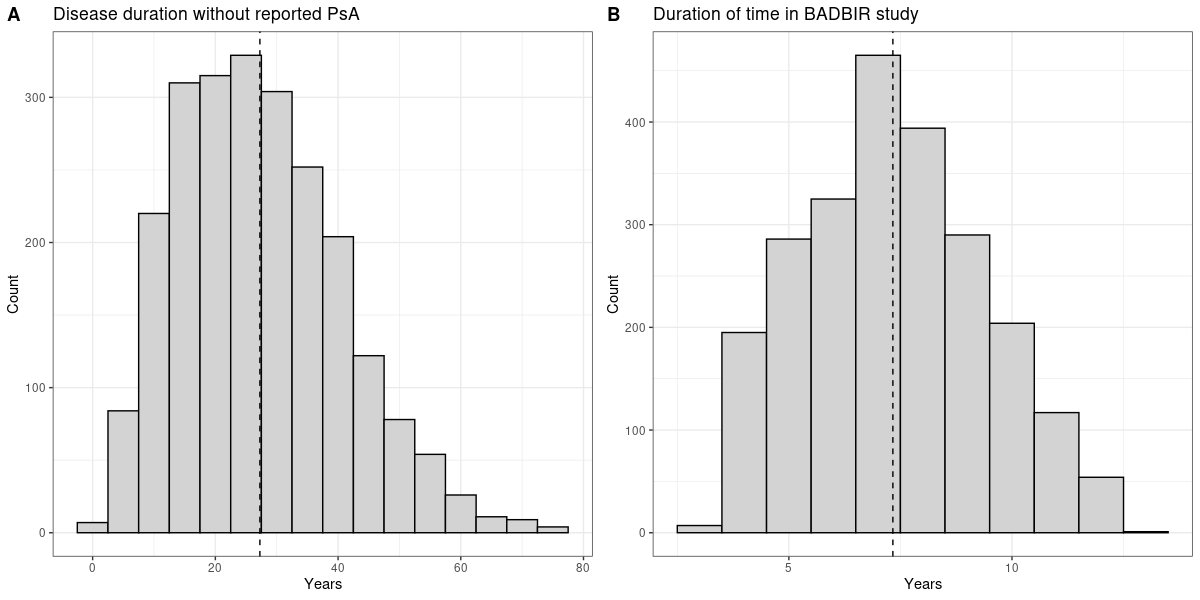


Supplementary Figure 1: summary of clinical data from the BSTOP cohort. All clinical data accrued within the BADBIR study is matched to BSTOP participants A) disease duration without recording a diagnosis of PsA and B) length of time as a participant of the BADBIR study for the cutaneous-only psoriasis patient group. Dashed vertical line indicates mean of the distribution.


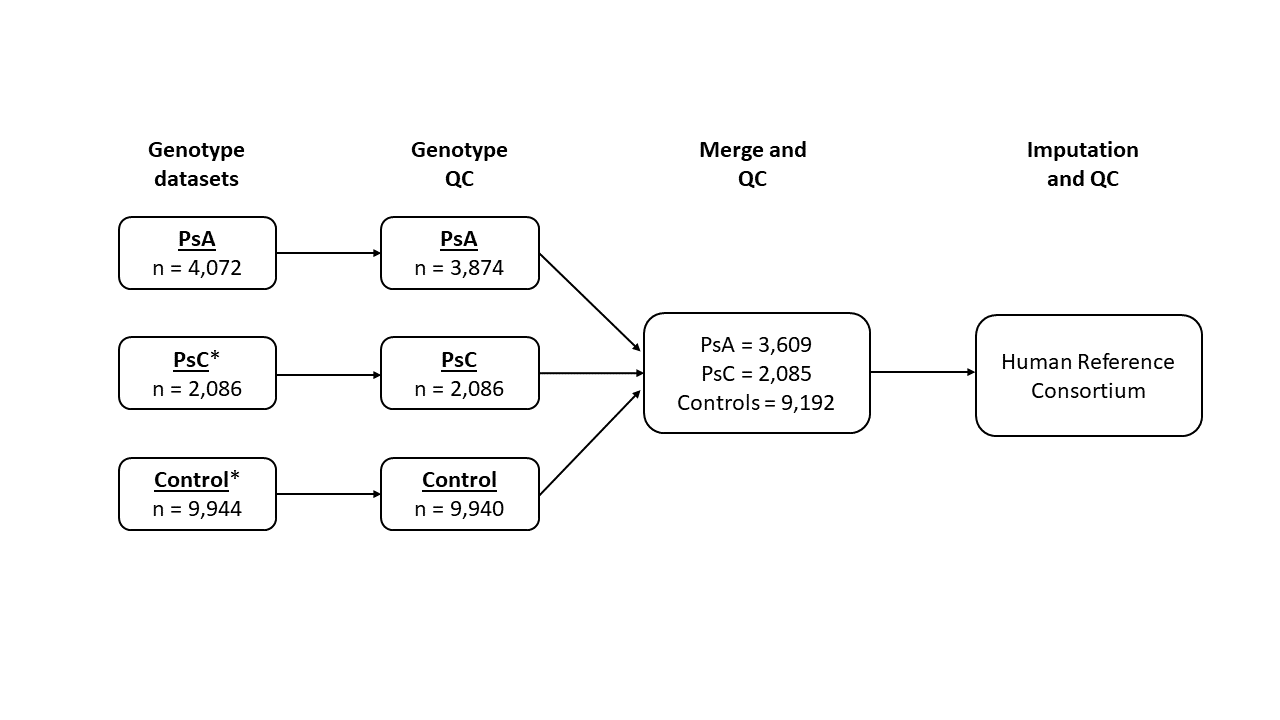


Supplementary Figure 2: genotyping and quality control of the PsA-BSTOP dataset. The three datasets were individually QC’d for removal of low-quality SNPs and sample (*datasets were received already QC’d). The three datasets were merged on an intersection of SNPs and further sample QC was performed to remove related individuals and outliers based on PCA. This combined dataset was used for imputation


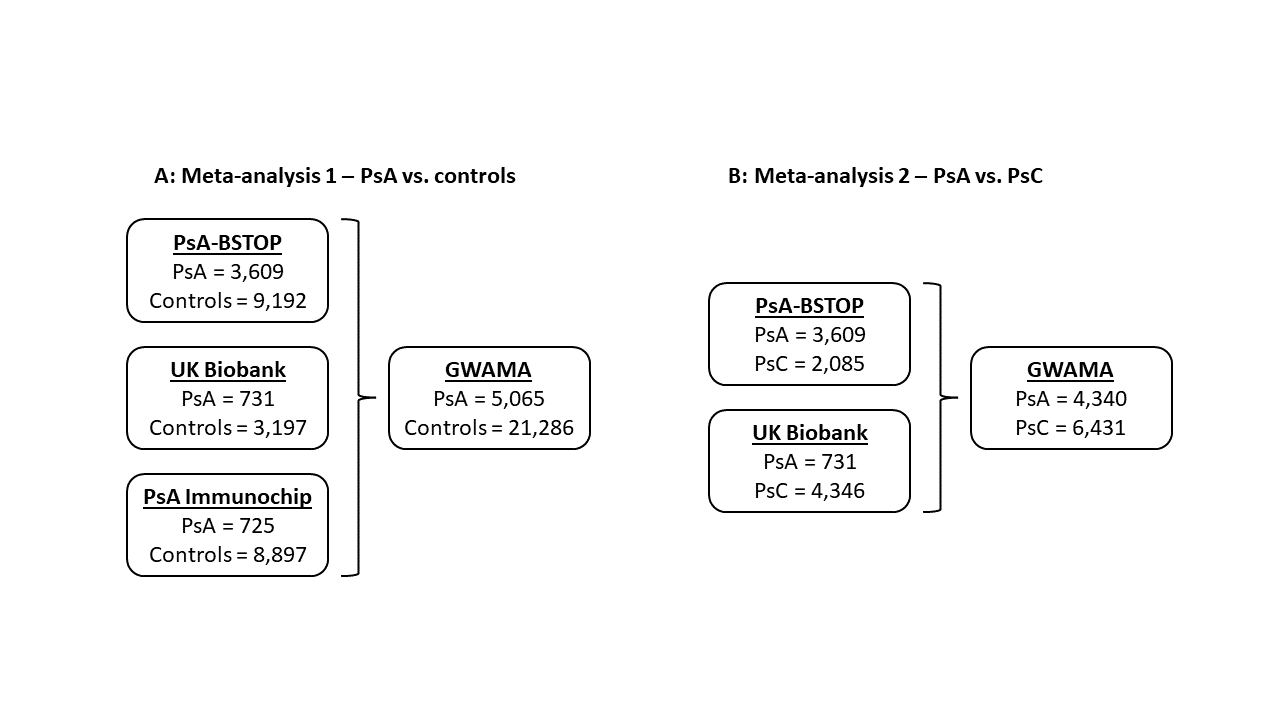


Supplementary Figure 3: summary of reported meta-analyses. A) meta-analysis of PsA vs. healthy control data was performed across three datasets excluding overlapping and related samples. B) Meta-analysis of PsA vs. PSC was performed over two datasets excluding overlapping and related samples.


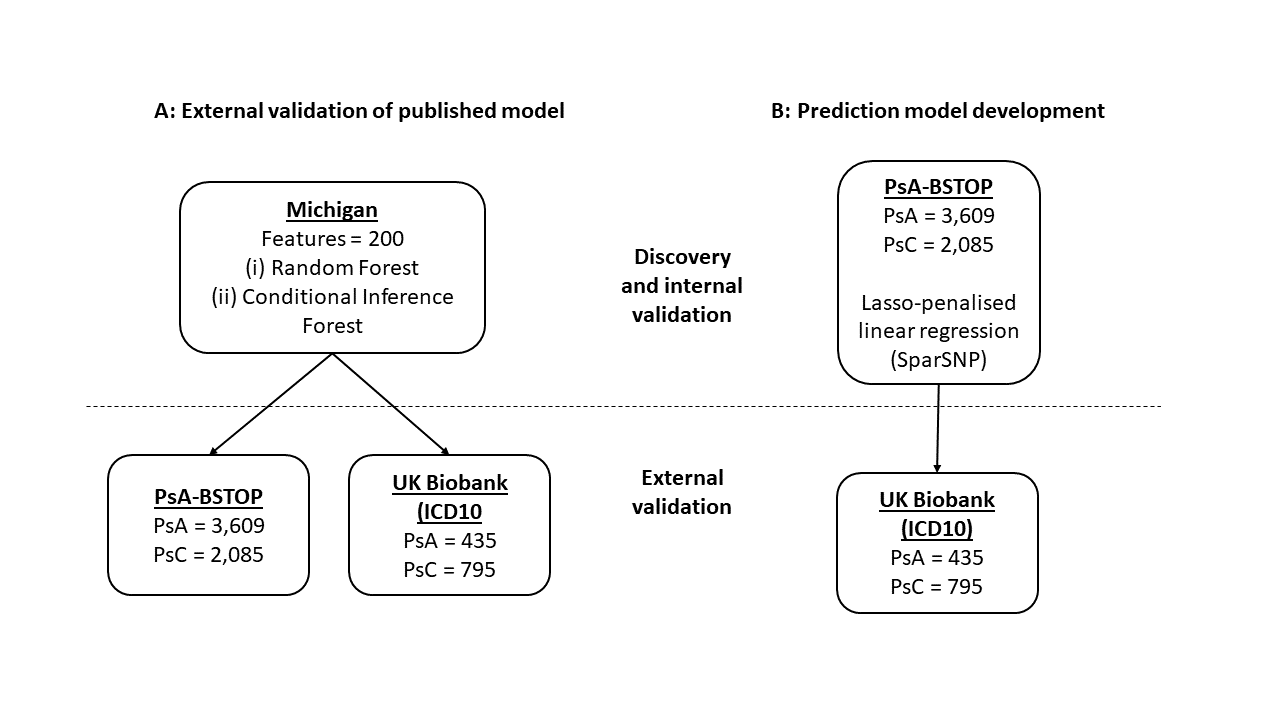


Supplementary Figure 4: summary of prediction analyses. A) external validation of the previously reported Michigan classification pipeline was attempted in two independent datasets. B) A newly created prediction model was externally validated in one independent dataset.


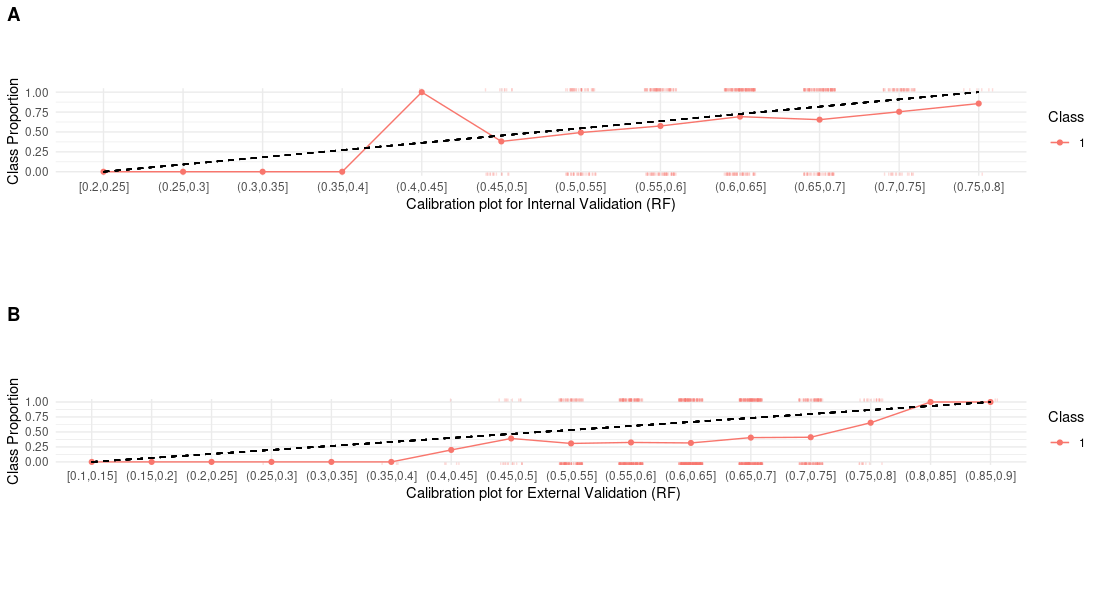


Supplementary Figure 5: calibration curve for the random forest model. A) internal validation using the PsA-BSTOP GWAS dataset and B) external validation using the UK Biobank ICD10 dataset.


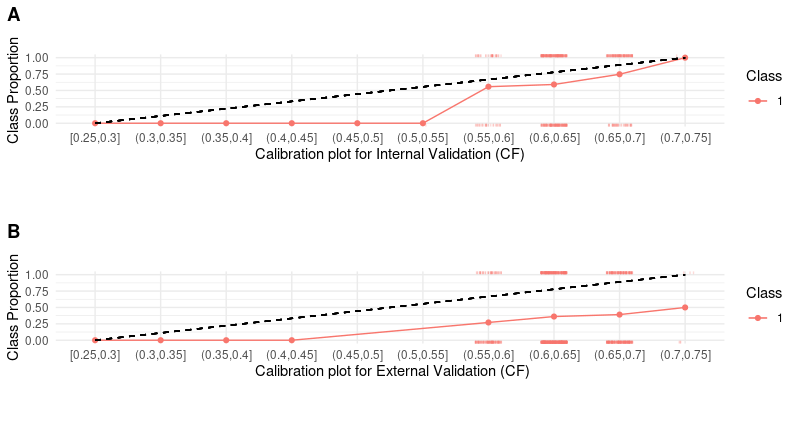


Supplementary Figure 6: calibration curve for the conditional inference forest model. A) internal validation using the PsA-BSTOP GWAS dataset and B) external validation using the UK Biobank ICD10 dataset.


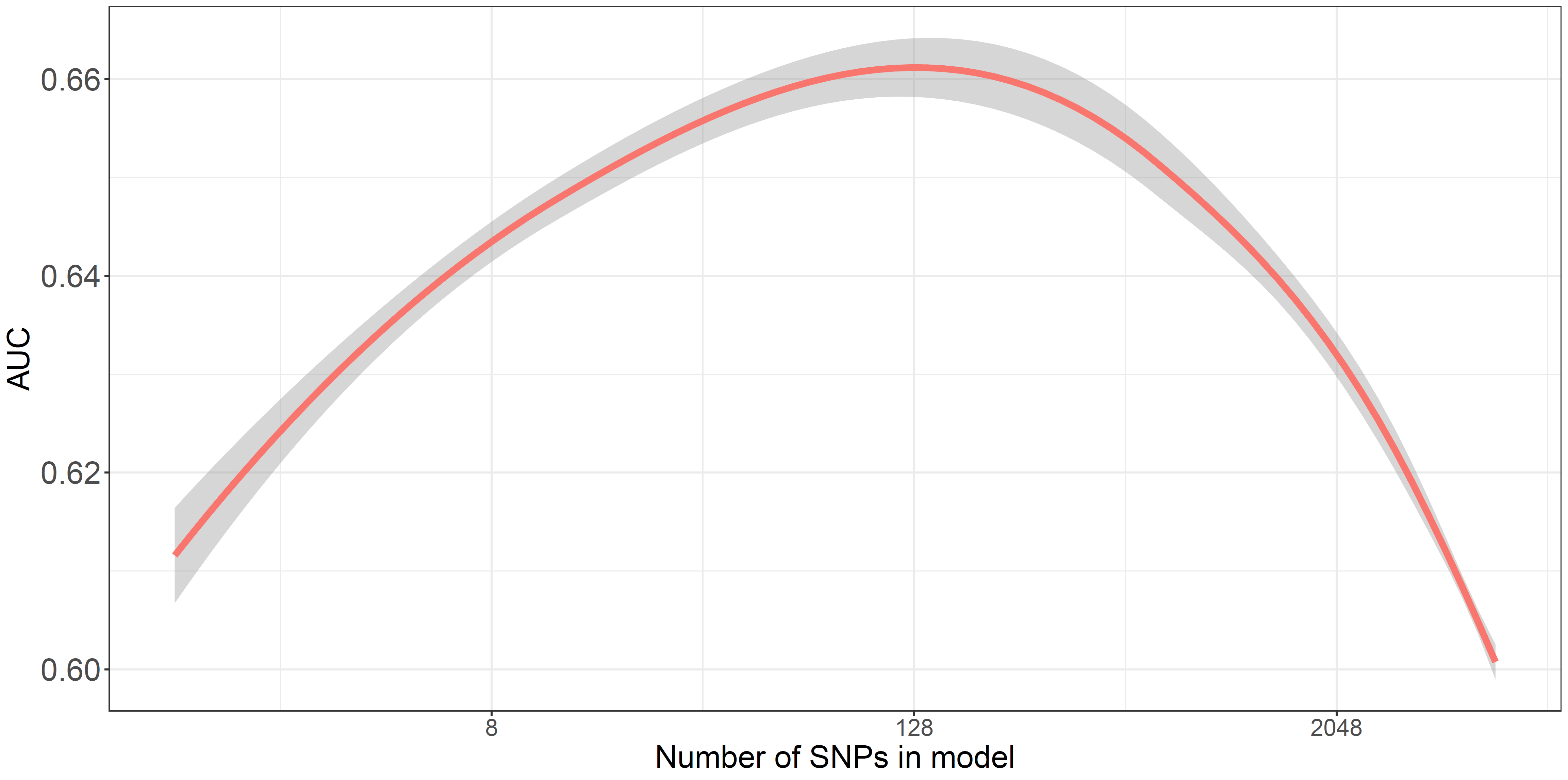


Supplementary figure 7: SparSNP AUC results. 10x10 Cross-validation in PsA-BSTOP training dataset. The best model based on maximum AUC contained 118 SNPs (AUC = 0.66).


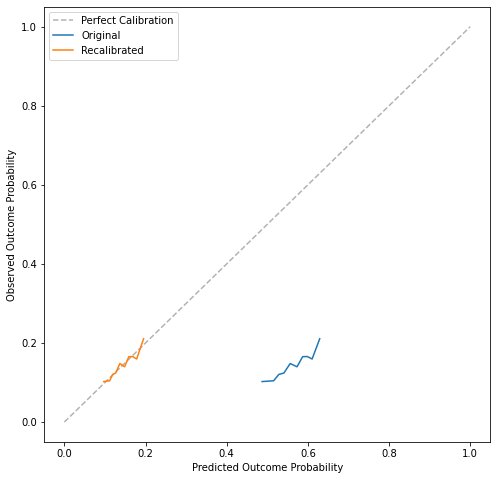


Supplementary figure 8: SparSNP calibration curve. Applied to the Biobank data. The predicted probabilities are divided into 10 quantiles, before plotting each quantile's mean predicted probability against its actual incident rate. The original calibration curve (Blue) shows poor calibration, with probabilities being greatly overestimated when compared to the actual rate of positives. After recalibrating the model for the new population, the curve (Orange) lays closer along the ideal (dotted diagonal). However, even after recalibration, the model clearly fails to predict high probabilities, which helps explain the low AUC.
